# Non-specific neck pain evaluation using functional linear models with the limma correction

**DOI:** 10.1007/s11517-025-03400-3

**Published:** 2025-07-08

**Authors:** Elisa Aragón-Basanta, Guillermo Ayala, Álvaro Page, Pilar Serra-Añó

**Affiliations:** 1 Instituto Universitario de Ingeniería Mecánica y Biomecánica, Universitat Politècnica de València, Camino de Vera s/n, Valencia, 46022 Spain; 2 Departamento de Estadística e Investigación Operativa, Facultad de Matemáticas, Universitat de Valéncia, Avda Vicent Andrés Estellés s/n, Burjassot, 46100 Valencia Spain; 3 Departamento de Fisioterapia, Universitat de Valéncia, Gascó Oliag 5, Valencia, 46010 Spain

**Keywords:** Limma, Functional data analysis, Neck disability index, Benjamini-Hochberg correction, Multiple linear regression

## Abstract

**Abstract:**

We have analyzed the relationship between disability and neck flexion-extension kinematics in non-specific neck pain subjects. A functional approach is used considering the angle, velocity, and acceleration curves. Different regression models have been fitted for each time in order to obtain these curves using scalar predictors such as the Neck Disability Index (NDI), age, sex, and neck length. In addition to classical regression, a limma (Linear Models for Microarray Data) model has been used, which improves the fit by modifying the estimation of the variances of the different fits using an empirical Bayes approach. As point-by-point adjustments are performed, this introduces a multiple comparison problem, and the corresponding *p*-values have to be adjusted in order to control the false discovery rate (FDR). In particular, a Benjamini-Hochberg method was used. The results show significant differences between raw and adjusted *p*-values for all variables, so spurious results were detected, e.g., the effect of neck length on velocity and acceleration curves. Differences between usual multiple linear regressions and the modified fits using the limma method (limma models) are minor, with a slight decrement of *p*-values in limma models. Once the *p*-values are adjusted, none of the variables analyzed significantly affects the angular curves. In contrast, NDI and age affect velocity and acceleration curves. Furthermore, the study of *p*-values throughout the movement shows that velocity and acceleration curves provide complementary information, so they should be used together in neck kinematics studies.

**Graphical abstract:**

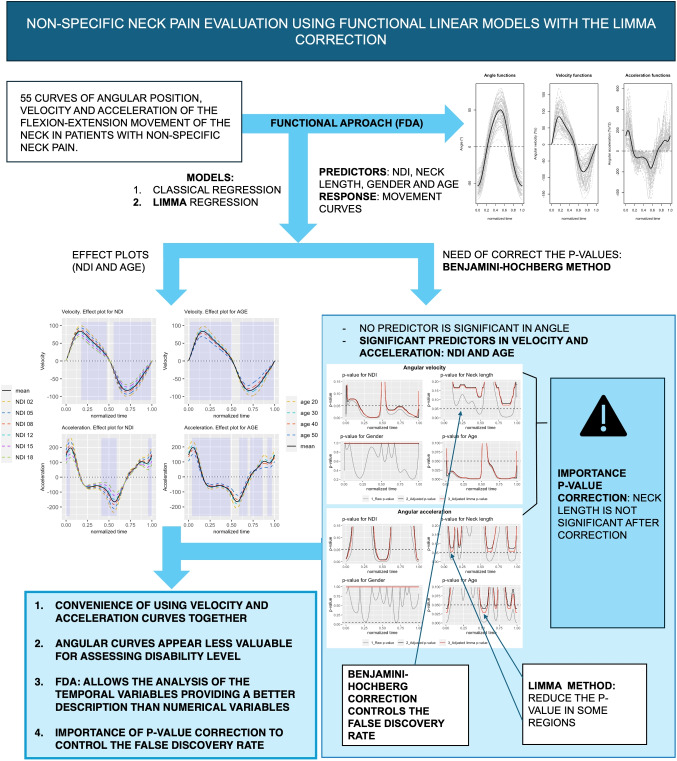

**Supplementary Information:**

The online version contains supplementary material available at 10.1007/s11517-025-03400-3.

## Introduction

Neck pain is a complex condition with multiple contributing factors and remains a major public health concern. It is the second most prevalent musculoskeletal pain disorder worldwide, with its incidence increasing significantly over the past three decades [1].

In clinical settings, its assessment usually relies on patient-reported outcome measures such as the Visual Analog Scale (VAS), the Neck Disability Index (NDI), and basic range of motion tests [2]. While these tools are easy to administer, they are subjective, sometimes insensitive to subtle functional changes, and lack temporal resolution. Therefore, there is a growing interest in objective, movement-based metrics that can provide more precise information on motor behavior and its alterations due to pain or dysfunction [3]. This has led to growing interest in objective, movement-based metrics that can offer more detailed insights into motor behavior and how it is affected by pain or dysfunction. Cervical kinematic analysis, in particular, has proven useful for evaluating injury severity and monitoring rehabilitation progress. Extensive research has explored the relationship between neck disorders and kinematic parameters, with recent reviews providing a comprehensive overview of the findings [4, 5]. Studies have consistently demonstrated an association between neck pain and reduced range of motion, movement velocity, and acceleration [6–10]

However, most published studies analyze changes in kinematic patterns by comparing people with diverse pathologies to healthy controls [11]. While this type of analysis can reveal general differences, it provides limited clinical insight, as the actual value of functional or kinematic evaluation lies in assessing the severity or progression of the condition. Despite this, studies examining these relationships remain scarce. Those studies exploring correlations between kinematic patterns and clinical indices (obtained from questionnaires or clinical tests) have reported weak associations in populations with neck pain [8, 12, 13].

So far, some reasons for these weak associations have been pointed out. On the one hand, there are limitations in the reproducibility of kinematic variables [14], generally lower than those of clinical indices or pain scales [15]. On the other hand, most published studies ignore the continuous nature of joint movements and reduce the kinematic curves to scalars like as ranges, maxima, minima, or event durations [16].

The movement of a joint can be represented by curves of positions, velocities, and accelerations during the entire range of motion, which the type and severity of the pathology can influence. From a mathematical point of view, these curves can be described and treated as functional variables within the framework of functional data analysis (FDA). This branch of statistics extends and generalizes the classical methods from numerical to functional variables [17]. The use of FDA techniques has essential advantages over the classical description of the movement since they maintain all the information contained in the curves (and in their derivatives) without reducing them to a set of numerical values (maxima, minima, event durations) that do not represent the relationships with motor coordination and dynamics of the movement. Two reviews of FDA applications in Biomechanics are given in [11] and [18]. Additionally, there are other approaches to work with continuous curves, such as Statistical Parametric Mapping, as used in [19].

Methods based on continuous signal analysis have shown greater sensitivity in detecting changes in movement patterns associated with pathologies [16, 20–22]. However, these results should be qualified for three reasons. First, using continuous curves instead of scalars implies an increase in the dimensionality of the problem, which means a risk of overfitting that must be taken into account. Two fundamental strategies to prevent this risk are reducing the dimensionality through function bases or using some regularisation procedure [17]. In any case, it is convenient to evaluate the models using some criterion, such as the cross-validation criterion (GCV) or the Akaike information criterion (AIC) [18].

Moreover, local inference is usually performed when comparing curves with the FDA, i.e., testing a null hypothesis for each point along the entire curve, which involves a large amount of hypothesis testings, producing large number of false positives [23]. Classical methods for multiple testing correction, such as Bonferroni correction, are too conservative for so many tests, making them unsuitable for FDA applications. Therefore, other corrections should be implemented, such as the Benjamini-Hochberg correction [24].

Finally, the values observed at different times are not independent, i.e., the different fits used related information and this has to be considered.

This paper has two main objectives. First, to analyze the relationship between neck kinematics and self-reported disability in individuals with non-specific neck pain, as measured by the Neck Disability Index (NDI) [25], using a functional approach. Instead of conventional scalar descriptors, we examine which specific portions of the kinematic curves (position, velocity, and acceleration) are associated with different levels of disability. This approach preserves the temporal structure and richness of the movement data.

The second objective is to address the increment of type I error rate associated with multiple comparisons in functional data analysis. To this end, we apply the limma model (Linear Models for Microarray Data), originally developed for high-dimensional gene expression analysis, as it offers regularization and multiple testing correction adapted to the complexity of functional data [26, 27]. Unlike the classical linear model, based on least squares fitting, the limma model uses empirical Bayesian regularisation, which helps stabilize the model estimates and incorporates a correction for multiple testing, complemented by the Benjamini-Hochberg correction [28]. These objectives aim to answer the following research questions: 1) Which specific regions of cervical kinematic curves are significantly associated with self-reported disability levels in individuals with non-specific neck pain?; and 2) how do the detected associations between cervical kinematics and disability (NDI) change when type I error is controlled through multiple comparison correction methods in functional data analysis?

## Methods

### Patients

The data on neck movement kinematics used in this study were initially collected to study the effect of a neck manipulation session on patients with non-specific neck pain [29]. Twenty-eight subjects participated, with measurements taken before and after treatment, resulting in 56 observations. The study involved kinematic tests and assessments of disability status. While the original analysis focused on conventional numerical variables such as ranges of movement and velocity, this study utilizes the complete movement data recorded. All participants signed an informed consent, and the study protocols were approved by the University of Valencia Ethics Committee (H1450106985729). All procedures were performed following the latest revision of the Declaration of Helsinki.

### Clinical and functional procedures

For the kinematic study, neck flexion-extension tests were conducted per a specified protocol [14]. Participants were seated in a chair that restricted trunk and leg movement, allowing only neck and head movement. This movement was recorded using a video-photogrammetry system with eight reflective markers on a headband. Participants were instructed to look at their eyes in a small mirror 2.5 m away at eye level to establish a reference position. An additional calibration measurement defined an anatomical reference system with markers on specific facial points removed after calibration.

During each measurement session, subjects performed cyclical neck flexion-extension movements at a comfortable maximum speed. Angles and velocities were calculated from the marker coordinates. The continuous records were divided into complete cycles, discarding the first and last cycles [30]. The time scale was normalized, and the functional mean of the five position and angular velocity curves was used as dependent functional variables.

The Neck Disability Index (NDI), a questionnaire that assesses functional ability on a 0–50 scale, was used as the independent variable [31]. The NDI questionnaire was administered before each measurement session to provide a numerical independent variable for each observation of the functional dependent variables. In addition, the weight, age, sex, and neck length of each patient, measured in the reference position, were recorded according to the procedure described in [32].

Therefore, as each subject performed two repetitions before and after treatment, there are 56 measurements. However, only 55 (35 women and 20 men) were included, as one had to be discarded due to errors during the measurement process.

### Data analysis

Let us choose a time *t*. We have predictors, denoted $\varvec{x}_i$, common for all the times and the **observed** response at this time, $y_i(t)$ where $i=1,\ldots ,n$ denotes the observation. We are interested in the conditional distribution of the **random** response $Y_i$ given the predictors $\varvec{x}_i$.

#### The limma model

More formally, our data set consists of scalar predictors and a functional response: $(\varvec{x}_i,y_i)$ with $i=1,\ldots ,n$ where $x_i \in \mathbb {R}^p$ and $y_i$ is a function defined on the interval [0, *T*]. We are going to consider a linear model for the data set $(\varvec{x}_i,y_i(t))$ for $i=1,\ldots , n$. We are going to assume that the random response $Y_i(t)$ can be modeled as

1 $$\begin{aligned} Y_i(t) = x_i^T \varvec{\beta }(t) + \epsilon _i(t), \end{aligned}$$

where the random errors $\epsilon _i(t)$ (for different *i*’s) are independent and identically distributed with a common normal distribution with null mean and variance $\sigma ^2(t)$, i.e., the usual multiple regression model.

A discrete set of times will be considered, $\{t_1,\ldots ,t_K\}$ within the time interval [0, *T*]. In order to remark its discrete nature we will denote $t_k$ instead of *t*.

Observations from different times are not independent and the number of times is much more larger than the sample size. This is common in statistical analysis of omics data where the features studied (genes, exons, probes, proteins, $\ldots$) used to be thousands and the number of observations a few tens. Additionally, the features (responses) are not independent. In this paper, we used a well-known method in the statistical omics literature, the **limma** method proposed in [26] and implemented in [27].

For each $t_k$, let us denote $\hat{\varvec{\beta }}(t_k) = (\hat{\beta }_1(t_k), \ldots ,\hat{\beta }_p(t_k))$ the maximum likelihood estimators of the vector of coefficients $\varvec{\beta }(t_k) = (\beta _1(t_k), \ldots , \beta _p(t_k))$. The unbiased estimator of $\sigma ^2(t_k)$ is denoted by $S^2(t_k) = \sum _{i=1}^n (Y_i(t_k) - x_i^T\hat{\varvec{\beta }}_j(t_k))^2/(n-p)$.

Using an empirical Bayes method, the limma approach borrows information from different fits (different times) to improve the estimation of $\sigma ^2(t_k)$. A prior distribution for $\sigma ^2(t_k)$ has to be specified where it is assumed that $1/\sigma ^2(t_k)$ follows a chi-squared distribution: $\frac{1}{\sigma ^2_k} \sim \frac{1}{d_0 s^2_0} \chi ^2_{d_0}$.

A prior distribution is assumed for the coefficients $\varvec{\beta }$. For the *j*-th coefficient $\beta _j(t_k)$ it will be assumed that it is not null with probability $p_j$. Note that this probability does not depend on $t_k$, i.e., $P(\beta _j(t_k) \ne 0) = p_j$, the proportion of the interval [0, *T*] where the *j*-th coefficient is not null. Really this proportion should be understood as the proportion of the times $t_k$. Furthermore, a normal distribution is assumed for the non null coefficients, $\beta _j (t_k) | \sigma ^2(t_k),\beta _j(t_k) \ne 0 \sim N(0, v_{0j}\sigma ^2(t_k))$. It was proved in [26] that the posterior mean of $1/\sigma ^2(t_k)$ is given by

$$\begin{aligned} E \left[ \left. \frac{1}{\sigma ^2(t_k)} \right| s^2(t_k) \right] = \frac{1}{\tilde{s}^2(t_k)}, \end{aligned}$$

with

$$\begin{aligned} \tilde{s}^2(t_k) = \frac{d_0 s^2_0 + d(t_k) s^2(t_k)}{d_0 + d(t_k)}. \end{aligned}$$

The moderated *t*-statistic is defined as

2 $$\begin{aligned} \tilde{T}_j(t_k) = \frac{\hat{\beta }_j(t_k)}{\tilde{S}^2(t_k) \sqrt{v_{jj}^{(t_k)}}}. \end{aligned}$$

It was proved that $\tilde{T}_j(t_k)$ and $S^2(t_k)$ are independent and, under the null hypothesis of null coefficient ($H_k: \beta _j(t_k) = 0$), the moderated *t*-statistic $\tilde{T}_j(t_k)$ has a *t*-Student distribution with $d(t_k) + d_0$ degrees of freedom ([26, sección 4]),

3 $$\begin{aligned} \tilde{T}_j(t_k) \sim t_{d(t_k) + d_0}. \end{aligned}$$

The hyperparameters $d_0$ and $s_0$ were estimated from the data and details can be found in [26]. We will be considered the hypothesis tests: $H_k: \beta _j(t_k) = 0$ vs $K_k: \beta _j(t_k) \ne 0$. The corresponding *p*-value is evaluated under the null distribution

4 $$\begin{aligned} p_j(t_k) = P \left( \left. \left| \tilde{T}_j(t) \right| > \tilde{t}_j(t_k) \right| H_k\right) , \end{aligned}$$

where $\tilde{t}_j(t_k)$ is the observed value of $\tilde{T}_j(t_k)$.

By using the equation (4), we have a function $p_j$ observed at the discrete times $t_k$’s. In fact, it could be defined a functional object using functional data analysis using these estimated values. The number of times used is chosen by the user and a large number of points could be chosen.

We have multiple testing problem because a test of null coefficient is performed at different times. The *p*-value in equation (4) will be called the **raw** *p*-value. If we use these *p*-values without any modification then the type I error or false positive rate is very large. A global error rate has to be chosen in order to control it. The usual false discovery rate (FDR) will be used. This is an error rate that controls, in our case, the proportion of false significant times multiplied by the proportion of significant times. It was defined in [28] and widely used in the omics literature. The raw *p*-values are adjusted in order to control the error rate (FDR in our case). In particular, two different procedures have been used: the well-known method of Benjamini-Hochberg [28] where the different raw *p*-values are assumed independent which is not true, and the method of Benjamini-Yekutieli where positive correlations between *p*-values are assumed [33].

#### Models

Three different functionals on scalar regression models have been fitted. The functional responses are angle, velocity, and angular acceleration respectively. The common predictors used have been the level of disability (NDI), age, neck length and gender.

Thus, a point-by-point regression model is carried out for each time $t_k$ of the previous curves. For each time $t_k$, we have a vector of coefficients corresponding to the predictors, $\varvec{\beta }(t_k)$. The estimation of the standard error of these coefficients has been performed using two procedures: the usual method (without using information between different times) and the limma model (where the different fits are used to estimate the variance of the random error). Corresponding with these two estimators we will have the usual *t*-statistics and the moderated *t*-statistics, and two *p*-values, one per statistic, for testing a null *j*-th coefficient at time $t_k$. In short, for the *j*-th scalar predictor we have two *p*-values that can be denoted $p_{uj}$ and $p_{lj}$ when the **usual** and **moderated** *t*-statistics are used for the *j*-th coefficient. For this paper, 100 points (tests) were considered. However, a study with different number of times has been done showing the robustness of the method with respect to the number of points. It is included in Supplementary Material 2.

For the *j*th coefficient, the *p*-values $p_{uj}(t_k)$ for different times $t_k$’s have to be corrected because a multiple testing problem appears with a strong correlation between close times. Two procedures of correction (or adjustment) have been chosen. The first one is the Benjamini-Hochberg correction. It is a kind of standard in the omics literature. However, independent *p*-values are assumed. This is not true in the omics but, particularly, it is not true in functional on scalar regression. It seems more suitable to use the Benjamini-Yekutieli correction where a positive dependence between *p*-values is assumed. The adjusted *p*-values will be denoted $\tilde{p}^{(BH)}_{uj}(t_k)$ ($\tilde{p}^{BH}_{mj}(t_k)$) corresponding to the usual *t*-statistic (respectively moderated *t*-statistic) for the *j*-the predictor at time $t_k$. The *p*-values $\tilde{p}^{(BY)}_{uj}(t_k)$and $\tilde{p}^{BY}_{mj}(t_k)$ are the analogous adjusted *p*-values using the Benjamini-Yekutieli correction. In this paper it will be shown the functions $\tilde{p}^{(BY)}_{uj}(t_k)$ and the corresponding $\tilde{p}^{BY}_{mj}(t_k)$ are included in the supplementary material.

The R programming language [34], the R packages fda [35], fda.usc [36], limma [37], and Biobase [38] packages have been used in the study. Note that limma and Biobase are from the project Bioconductor [39].

## Results

**Fig. 1:**
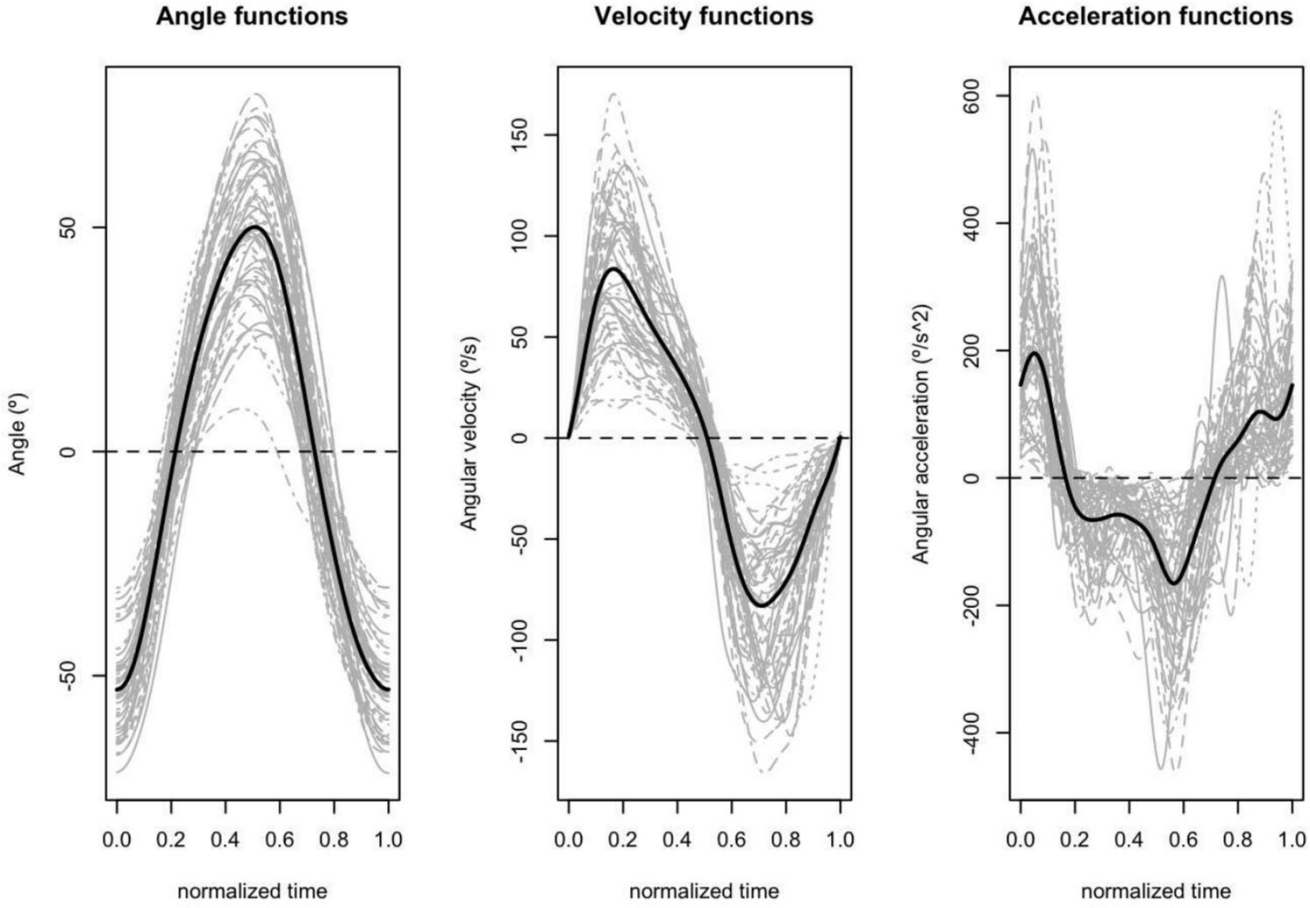
(Left) angle, (center) velocity, and (right) acceleration curves for different individuals (gray line) and the mean curve (black line)

Some numerical descriptions of the subjects who participated in the study are displayed in Table 1. In addition, the angle, velocity, and acceleration curves are displayed in Fig. 1, with the 55 curves in grey and the functional mean (in black). Table 2 displays the maximum, minimum, and range values of these curves.

**Table 1:** Mean and standard deviation of scalar variables measured in the study: disability index (NDI), age, neck length (cm), weight (kg), and height (cm)

Variable	Women	Men	Total
	*N* = 35 (63.6%)	*N* = 20 (36.4%)	*N* = 55
	Mean (sd)	Mean (sd)	Mean (sd)
NDI	11.11 (4.94)	8.35 (3.96)	10.11 (4.76)
Age	35.46 (12.21)	33.3 (13.06)	34.67 (12.45)
Neck length (cm)	14.36 (1.84)	16.08 (1.13)	14.98 (1.81)
Weight (kg)	65.06 (9.99)	83.58 (11.59)	71.79 (13.82)
Height (cm)	162.69 (6.80)	178.8 (7.15)	168.55 (10.41)

Three linear models were fitted at each time $t_k$ corresponding with three different responses: angle, angular velocity, and angular acceleration respectively. The predictors, common for the three models, were the level of disability (NDI), the length of the neck, the gender, and the age.

**Fig. 2:**
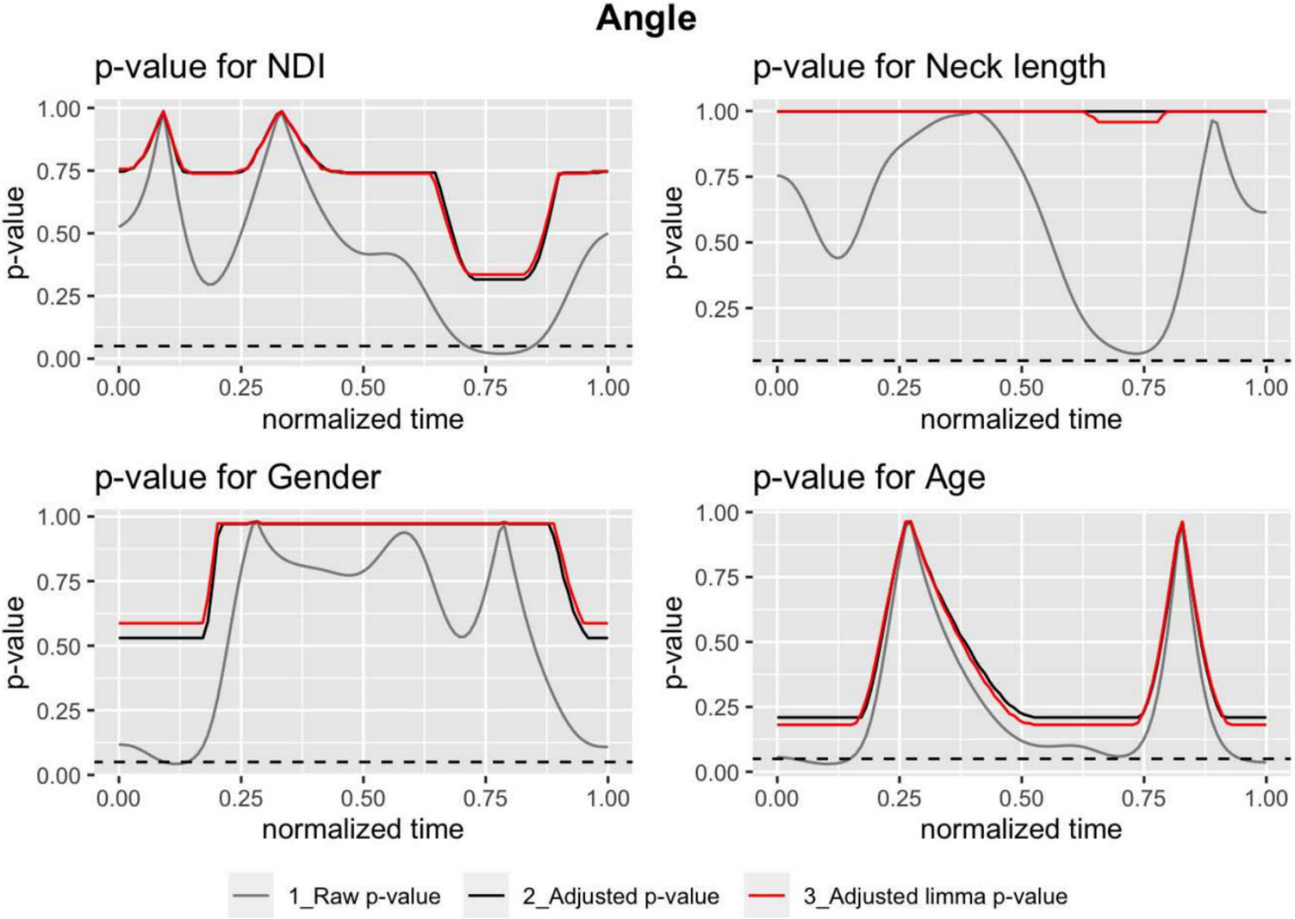
The *p*-value functions where the angle is the response. We have three functions per plot: the *p*-values using the *t*-statistic without any correction $p_{uj}(t_k)$ (grey); $\tilde{p}^{(BH)}_{uj}(t_k)$, the analogous function these *p*-values adjusted using the Benjamini-Hochberg correction and $\tilde{p}^{(BH)}_{lj}(t_k)$ the *p*-values with the limma method and the Benjamini-Hochberg correction (red). The predictor *j* corresponds to NDI (top-left), neck length (top-right), gender (bottom-left), and age (bottom-right). The dashed line corresponds to a constant value of 0.05. When the *p*-value function is lesser than this horizontal line then it could be considered significant. Note that for unadjusted (raw) *p*-values should be interpreted as a level of significance and for adjusted *p*-values the level of the false discovery rate

**Fig. 3:**
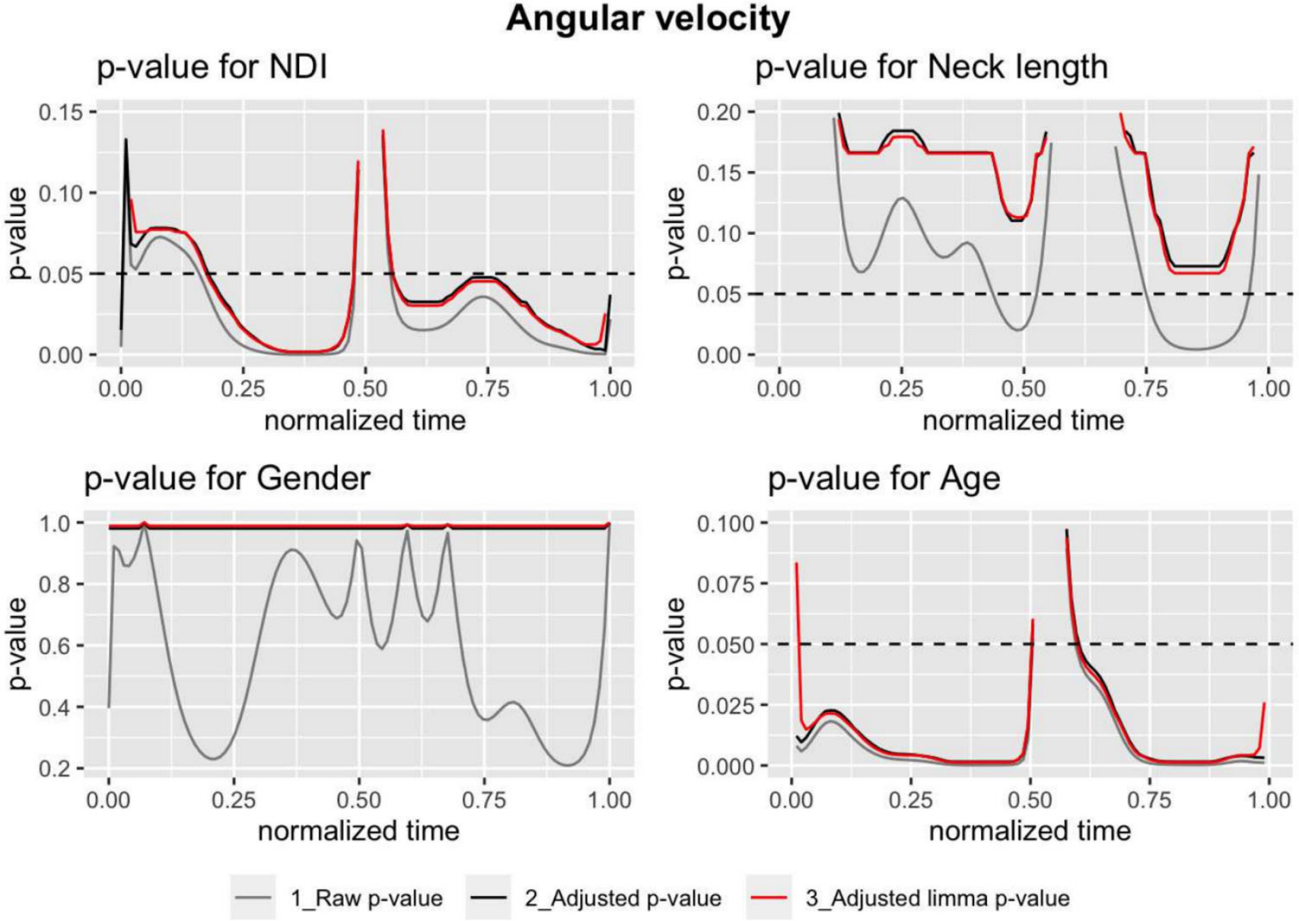
The *p*-value functions for velocity as response. See a detailed explanation in Fig. 2

**Fig. 4:**
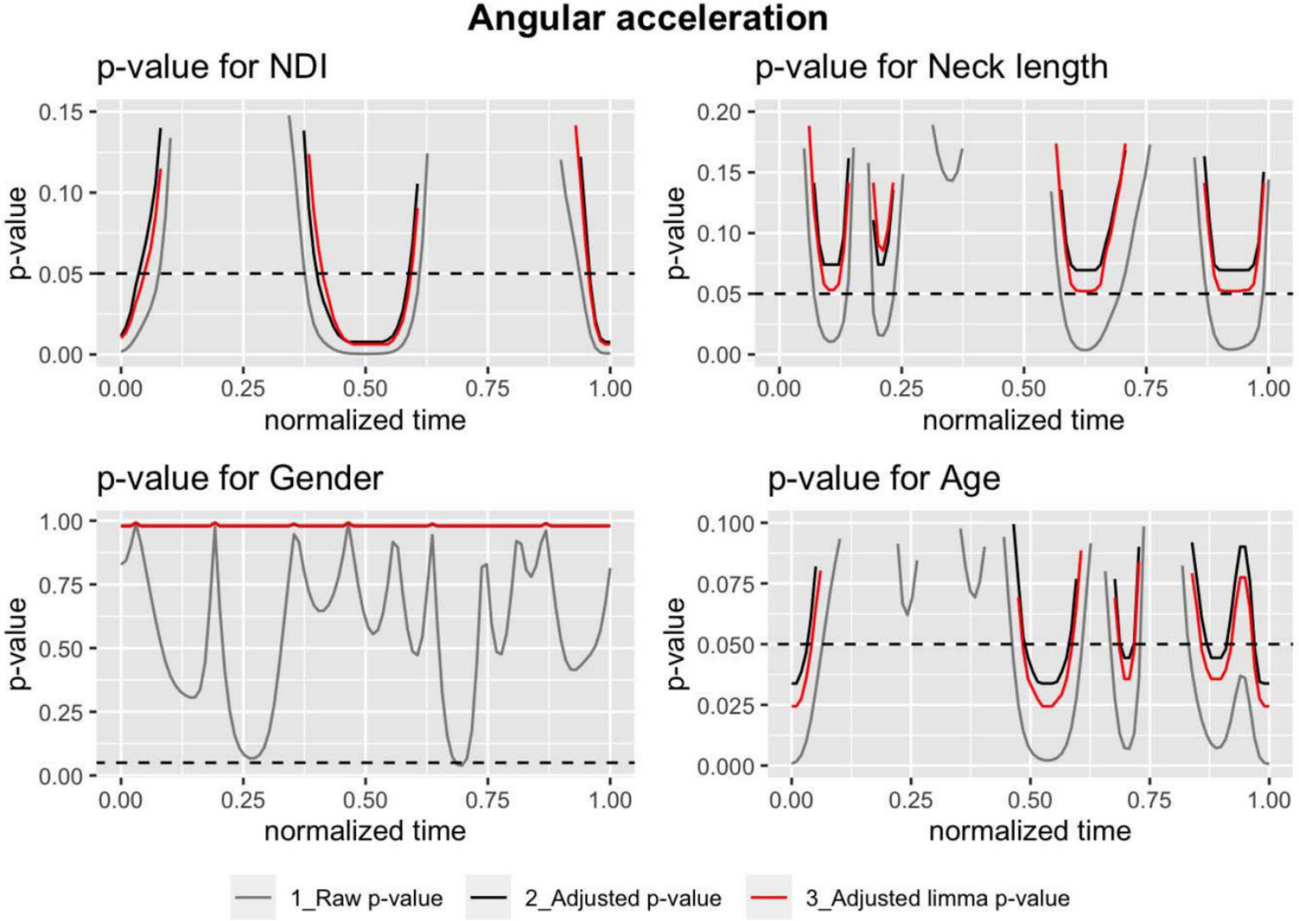
The *p*-value functions for acceleration as response. See the detailed explanation in Fig. 2

Figures 2, 3, and 4 display the *p*-values for different models, predictors and *p*-values. In each plot, the raw *p*-values without limma model (grey) are compared with those obtained from them by applying the Benjamini-Hochberg correction (black) and finally with the corrected *p*-values obtained using the limma model (red). A dashed black horizontal line, corresponding to a *p*-value equal to 0.05, has been added to identify in which areas of the movement the effect of each variable can be considered significant. The figure shows the results for a model that considers 100 points. The model was also implemented considering 1000 and 10,000 points, with almost no differences in the *p*-values obtained. These results can be found in Supplementary Material 2.

**Table 2:** Mean (standard deviation), maximum, minimum, and range values for the range of motion (RoM), range of velocity (RoV), and range of acceleration (RoA)

	RoM ($^{\circ }$)	RoV ($^{\circ }/s$)	RoA ($^{\circ }/s^2$)
Mean (sd)	104.19 (21.4)	176.37 (69.73)	423.3 (246.83)
Maximum	143.81	335.78	1064.18
Minimum	39.96	37.54	57.65
Range	103.85	298.25	1006.54

The Benjamini-Hochberg correction clearly increases the raw *p*-value functions. This change in *p*-values has a profound impact, making that, initially significant variables, such as neck length in the velocity model (Fig. 3) or acceleration model (Fig. 4), became no longer significant after the correction. Additionally, the limma regression modified the raw *p*-values of the usual regression models. It is helpful to share information between the fits in order to improve the estimation of the variance of the random error.

**Fig. 5:**
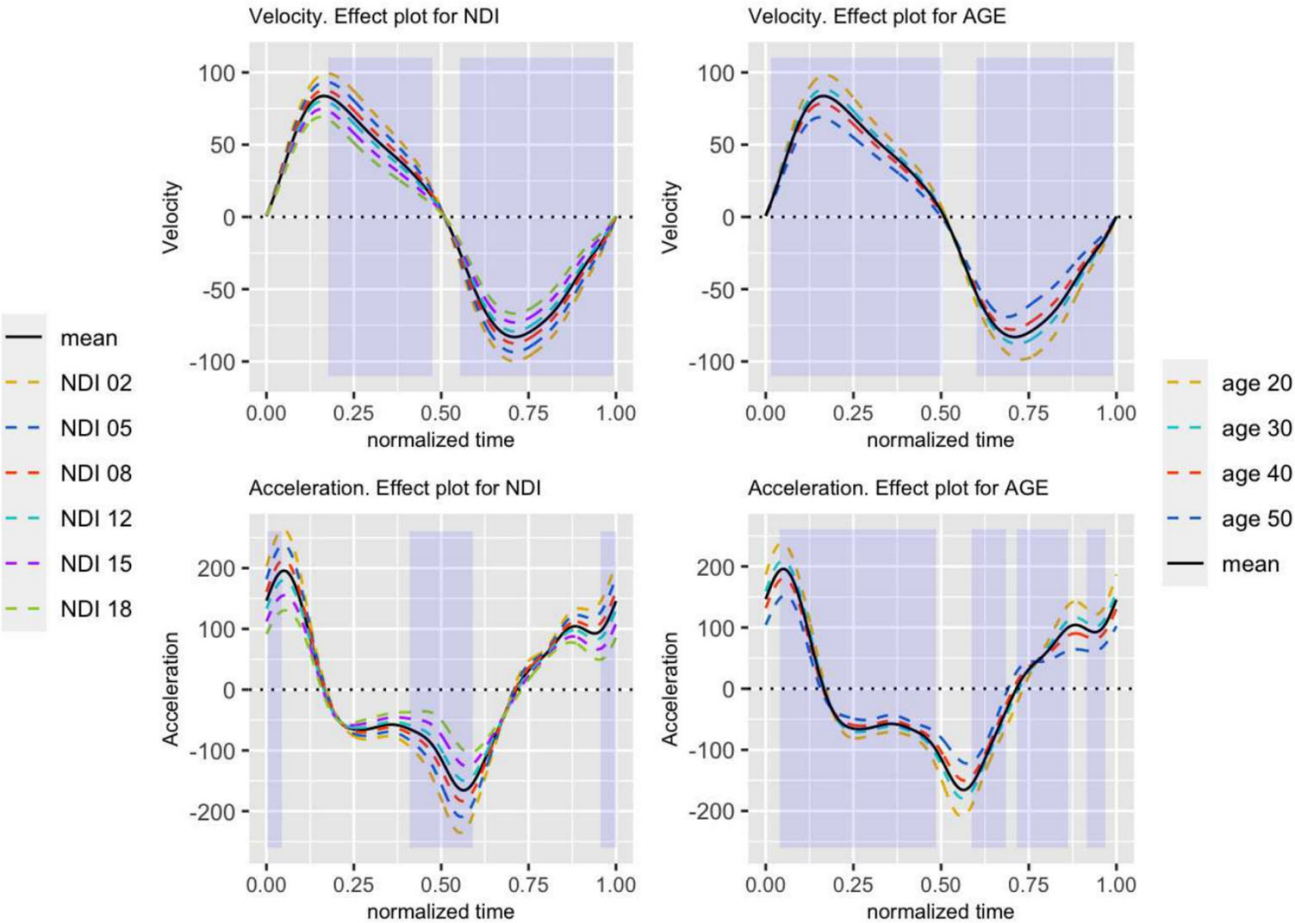
Effects plots for velocity (top) and acceleration (bottom) model. Left: effects plot for different NDI values, keeping constant the mean values for age, neck length, and gender shown in Table 1. Right: effects plots for different age values, keeping constant the mean values for NDI, neck length, and gender shown in Table 1. The legend of the effects plots for the different NDI values are on the left, while those for age are on the right. Areas of the movement where the effects are significant, according to the adjusted *p*-values, have been shaded in blue

Figure 5 shows the estimated effects (the estimated means) by the model for different values of NDI and age on the velocity and acceleration curves. The other predictors (gender, neck length) have no significant influence on the response although remain in the models. The effects on the flexion-extension angle curves have also not been represented since no significant relationship has appeared with any predictor variable once the adjustments on the *p*-values were made.

As can be seen, an increase in NDI leads to a decrease in the range of angular velocity due to a reduction in both the maximum and minimum values.

An increment of the age produces the same effect. The model predicts slower movements for older subjects.

These effects are similar in the case of the acceleration curves, where increments in NDI and age are associated with a decrement in the amplitude of the relative maxima and minima. However, the area where this influence is significant (shaded band in blue) is narrower than in the case of angular velocity.

It should be noted that the type of correction applied to the *p*-values does not affect the model coefficients or the effects shown in Fig. 5.

## Discussion

Neck kinematics can offer valuable insights into the severity of neck injuries. Although kinematic analysis can enhance our understanding of pathological progression and its impact on motor behavior, most studies focus primarily on comparing differences between healthy and diseased groups [9]. Fewer studies have explored correlations between kinematic variables and clinical scales, typically revealing weaker associations than between healthy and diseased subjects. These studies often examine discrete variables extracted from movement curves, such as maxima, minima, or ranges, overlooking the continuous nature of movement. Using the entire curve could yield better models [16]. However, such analyses should be approached cautiously due to the risk of spurious relationships arising from multiple comparisons and the high dimensionality of the data.

In this study, a linear regression model was used to analyze the effect of disability level on movement curves in patients with non-specific neck pain. The Neck Disability Index was employed as a predictor and control variable for individual characteristics such as sex, age, and neck length. The dependent variables included curves of angular displacement, angular velocity, and angular acceleration during cyclic and continuous flexion-extension movements. Each curve was represented by 100 points on a normalized time scale and fitted point by point to the model, giving *p*-value curves, *p*(*t*), which were corrected to reduce the risk of type I errors due to multiple comparisons.

Given many contrasts, classical *p*-value correction methods, such as the Bonferroni adjustment, are too conservative. Instead, the Benjamini-Hochberg method is recommended, as it allows control of the false discovery rate. This method is beneficial as it reduces the risk of incorrectly rejecting a true null hypothesis, which is a common issue in multiple comparisons. The limma regression has been used alongside classical regression, as it improves model fit by employing empirical Bayesian regularization, which enhances error variance estimation and stabilizes model corrections.

The results of these adjustments underscore the dramatic increase in the raw *p*-values when the Benjamini correction is applied. This increase causes predictors that were initially significant in certain areas of the curves to lose significance after correction (see Figs. 2, 3, and 4). This highlights the critical necessity of *p*-value correction in analyses of movement curves and emphasizes the risk of type I errors. The differences between the *p*-values corrected by the linear regression models and those corrected by the limma model are smaller. In most cases, the correction of the limma model is slightly less conservative than that of the linear regression model.

In the case of the angle curves, none of the predictor variables studied are significant after correction, while NDI and age are significant for velocity and acceleration. This result is consistent with previous studies, where very weak correlations have been found between NDI and range of motion [8, 12]. Moreover, it is essential to note that the Neck Disability Index (NDI) extends beyond the angular movement restriction assessment to evaluate factors such as strength, fatigue, and overall functional capacity, including movement speed and coordination. These aspects can be significantly impacted by non-specific pain, sometimes more so than the restriction of movement itself, which would account for the observed higher sensitivity in velocity and acceleration curves [10].

The *p*(*t*) functions corresponding to the velocity and acceleration curves show a significant relationship with NDI and age, both in the raw values and, to a lesser extent, in the corrected ones. Neck length affects velocities and accelerations when using the raw *p*-values but not after correction. Gender is not significant either before or after correction.

The influence of NDI and age on the velocity and acceleration curves indicate a decrease in the speed of movements as the NDI or the age of the patients increases. Thus, Fig. 5 shows that the higher the level of disability, the lower the speed and acceleration. Likewise, as age increases, the extreme values of both variables also decrease. These results corroborate those obtained in previous studies where ranges or maximum values of velocities and accelerations have been used [10, 40–43].

The effect of age and NDI on kinematic curves is illustrated in Fig. 5 (right), showing how velocity and acceleration curves change depending on patient age or NDI score. These curves represent the model’s fit, which does not rely on the method for correcting *p*-values. The correction method only affects error estimation without influencing model coefficients or the estimated effect size.

Regarding the effect of age on velocity, the model predicts slower movements as patient age increases. A similar trend is observed for acceleration, where older patients exhibit lower accelerations. This reduction is particularly pronounced at the movement extremes (maximum flexion and extension), where acceleration typically reaches its peak values. These findings align with previous studies [40, 43], which analyzed peak values rather than full kinematic curves. The observed reduction in movement speed, affecting both velocity and acceleration, has been linked to degenerative changes associated with aging and alterations in visual, vestibular, and neuromuscular function, which influence preferred movement speed [43].

A similar trend is observed in the effect of NDI on kinematic curves. A higher NDI score is associated with slower movements, implying lower velocity and acceleration across the entire movement range, particularly at local maxima and minima. These results are consistent with previous studies reporting a negative correlation between NDI and peak velocities in various pathologies [8, 10, 41, 42], though none have analyzed the effect of NDI on the complete curve. No studies examined the correlation between NDI and acceleration. However, Baydal et al. [40] reported a significant reduction in peak acceleration in whiplash patients compared to healthy controls.

Using continuous variables instead of peak values allows identifying specific regions in the curves where a predictor has the most significant influence, as highlighted by the shaded significance areas in Fig. 5. For NDI, two distinct regions significantly affect both velocity and acceleration curves (Fig. 5, left). In velocity, significant NDI influence appears in the deceleration phase of both extension and flexion movements. These results align with Aragón et al. [16], who performed a functional regression using flexion-extension velocity as a functional predictor for NDI estimation. The significant NDI influence is observed at the movement range extremes for acceleration.

This study found no significant relationships between NDI and angular curves across the entire range of motion, including peak flexion and extension. These findings suggest that velocity and acceleration curves provide more informative metrics for assessing neck disability than angular curves. This result aligns with previous studies, which have demonstrated that angular velocities are more closely related to functional impairments and neck pain than the range of motion (ROM) [16, 41, 42].

From a purely mechanical perspective, there is a simple reason why velocity curves could be more sensitive than position curves to changes associated with reduced cervical mobility. In cyclic neck movements, velocity amplitude depends on both movement amplitude (related to RoM) and execution time T, which is not explicitly represented when using a normalized time scale. For instance, in a harmonic movement with an angular curve amplitude A, velocity amplitude is proportional to A/T. Since patients with neck pain exhibit reduced RoM and slower movements [8], this effect should be more pronounced in velocity than in position curves. In other words, velocity curves capture movement amplitude and speed variations, whereas angular curves do not.

Additionally, reduced velocity has been linked to neuromuscular impairments, including decreased torque generation in neck muscles, reduced proprioception, and altered neck coordination [13]. Moreover, Devecchi [10] identified velocity as the movement characteristic most associated with fear of pain. However, their study did not measure acceleration or employ functional data analysis (FDA) methods for curve assessment.

The relationship between increased NDI and reduced acceleration has been scarcely investigated in cervical kinematics. Baydal [40] reported that whiplash patients exhibit lower peak acceleration and impaired movement harmony. In our study, NDI primarily affects acceleration at movement extremes, where acceleration reaches its highest values—specifically, during braking before the maximum extension (blue band around the normalized time 0.5 in Fig. 5) and subsequent acceleration at maximum flexion (normalized time 0 and 1.0). From a mechanical standpoint, slower periodic motion is inherently associated with lower acceleration amplitude. Additionally, the neuromuscular basis of this reduced acceleration—implying lower velocity amplitude—may stem from diminished muscle capacity to generate torque, particularly at movement extremes where acceleration peaks and maximal torque are required for deceleration and acceleration.

From a clinical perspective, our findings support the inclusion of velocity- and acceleration-based variables in the functional assessment of the cervical spine. These kinematic variables may offer greater sensitivity for detecting early or subtle impairments in motor control. While traditional motion analysis has relied on video-based systems, recent advances in wearable technologies—such as inertial measurement units (IMUs) [44] and virtual reality headset [45] offer more affordable and portable alternatives. These developments could facilitate the integration of dynamic kinematic assessment into clinical practice, although further progress is needed before widespread adoption becomes feasible.

One limitation of this study is the relatively small sample size, which could affect the generalization of the findings. However, this sample size is comparable to previous studies analyzing the relationship between neck pain and NDI during continuous movements. Despite this limitation, the results show significant associations between NDI and angular velocity and age and acceleration curves, with *p*-values *p* < 0.05 and *p* < 0.01, respectively, even after limma correction. However, the sample size could have influenced the lack of significance in the flexion-extension angle, which is consistent with previous studies that found a stronger relationship between disability and velocity than with range of motion. More extensive studies are needed to validate these findings or extend them to other pathologies.

It is usual to work with small sample sizes in this context. Obviously, a small sample size corresponds with a low statistical power. The limma method provides a better estimation of the variance of the random error by borrowing information between the different fits. These better estimations correspond obviously with a higher power.

## Conclusions

Functional data analysis (FDA) enables the investigation of associations between movement patterns and self-reported neck disability. By accounting for the whole temporal structure of motion curves, the functional approach can reveal relationships that may be diluted or missed when relying solely on scalar descriptors.

Our findings suggest that velocity and acceleration profiles may offer more sensitive indicators of cervical function than traditional range-of-motion (RoM) metrics, even in individuals with mild neck pain. Identifying specific movement phases affected by increased NDI, particularly during acceleration and braking rather than at peak extension, underscores the potential of these parameters to detect subtle motor control impairments.

Moreover, using *p*-value correction methods helps mitigate the risk of inflated type I error due to multiple testing, reinforcing the statistical robustness of our findings. This combination of functional modelling and rigorous statistical control offers a valuable framework for investigating the relationship between neck kinematics and disability.

Although further research is needed to define clinical thresholds, these results may support the development of improved functional assessment protocols and contribute to more precise monitoring of rehabilitation outcomes.

## Supplementary Information

Below is the link to the electronic supplementary material.

Supplementary file 1 (pdf 654 KB)

## References

[1] Cieza A, Causey K, Kamenov K, Hanson SW, Chatterji S, Vos T (2020) Global estimates of the need for rehabilitation based on the global burden of disease study 2019: a systematic analysis for the global burden of disease study 2019. The Lancet. 396(10267):2006–2017. 10.1016/s0140-6736(20)32340-0 10.1016/S0140-6736(20)32340-0 PMC7811204 33275908

[2] Misailidou V, Malliou P, Beneka A, Karagiannidis A, Godolias G (2010) Assessment of patients with neck pain: a review of definitions, selection criteria, and measurement tools. 9(2):49–59 10.1016/j.jcm.2010.03.002 10.1016/j.jcm.2010.03.002 PMC2943658 21629550

[3] Soltanabadi S, Vatandoost S, Lukacs MJ, Rushton A, Walton DM (2024) Association between clinical biomechanical metrics of cervical spine function and pain or disability in people with neuromusculoskeletal neck pain: protocol for a systematic review and planned meta-analysis. PLoS ONE 19(5):0303365. 10.1371/journal.pone.0303365 10.1371/journal.pone.0303365 PMC11086898 38728246

[4] Luc A, Tamer S, Hage R, Detrembleur C, Pitance L (2022) Do the kinematics and sensorimotor control of people with chronic non-specific neck pain differ from those of healthy individuals when assessed in an immersive virtual reality environment? A systematic review. Phys Ther Rev 27(6):430–443. 10.1080/10833196.2022.2143211

[5] Moghaddas D, Zoete RMJ, Edwards S, Snodgrass SJ (2019) Differences in the kinematics of the cervical and thoracic spine during functional movement in individuals with or without chronic neck pain: a systematic review. Physiotherapy 105(4):421–433. 10.1016/j.physio.2019.01.007 31005251 10.1016/j.physio.2019.01.007

[6] Stenneberg MS, Rood M, Bie R, Schmitt MA, Cattrysse E, Scholten-Peeters GG (2017) To what degree does active cervical range of motion differ between patients with neck pain, patients with whiplash, and those without neck pain? A systematic review and meta-analysis. Arch Phys Med Rehabil 98(7):1407–1434. 10.1016/j.apmr.2016.10.003 27984030 10.1016/j.apmr.2016.10.003

[7] Sarig Bahat H, Chen X, Reznik D, Kodesh E, Treleaven J (2015) Interactive cervical motion kinematics: sensitivity, specificity and clinically significant values for identifying kinematic impairments in patients with chronic neck pain. Man Ther 20(2):295–302. 10.1016/j.math.2014.10.002 25456272 10.1016/j.math.2014.10.002

[8] Treleaven J, Chen X, Sarig Bahat H (2016) Factors associated with cervical kinematic impairments in patients with neck pain. Man Ther 22:109–115. 10.1016/j.math.2015.10.015 26585294 10.1016/j.math.2015.10.015

[9] Franov E, Straub M, Bauer CM, Ernst MJ (2022) Head kinematics in patients with neck pain compared to asymptomatic controls: a systematic review. BMC Musculoskeletal Disorders. 23(1). 10.1186/s12891-022-05097-z 10.1186/s12891-022-05097-z PMC8848642 35172799

[10] Devecchi V, Alalawi A, Liew B, Falla D (2022) A network analysis reveals the interaction between fear and physical features in people with neck pain. Sci Rep 12(1). 10.1038/s41598-022-14696-8 10.1038/s41598-022-14696-8 PMC9253153 35787648

[11] Ullah S, Finch CF (2013) Applications of functional data analysis: a systematic review. BMC Med Res Methodol 13(1). 10.1186/1471-2288-13-43 10.1186/1471-2288-13-43 PMC3626842 23510439

[12] Howell E (2011) The association between neck pain, the neck disability index and cervical ranges of motion: a narrative review. J Can Chiropr Assoc 55:211–21 21886283 PMC3154067

[13] Tsang SMH, Szeto GPY, So BCL, Lau RWL, Tai JJ (2022) Using cervical movement velocity to assist the prediction of pain and functional recovery for people with chronic mechanical neck pain. Clin Biomech 93:105607. 10.1016/j.clinbiomech.2022.105607 10.1016/j.clinbiomech.2022.105607 35245780

[14] Venegas W, Inglés M, Page A, Serra-Añó P (2020) Paths of the cervical instantaneous axis of rotation during active movements–patterns and reliability. Med Biol Eng Comput 58(5):1147–1157. 10.1007/s11517-020-02153-5 32193862 10.1007/s11517-020-02153-5

[15] Jorritsma W, Dijkstra PU, Vries GE, Geertzen JHB, Reneman MF (2012) Detecting relevant changes and responsiveness of neck pain and disability scale and neck disability index. Eur Spine J 21(12):2550–2557. 10.1007/s00586-012-2407-8 22752592 10.1007/s00586-012-2407-8 PMC3508212

[16] Aragón-Basanta E, Venegas W, Ayala G, Page A, o, P (2024) Relationship between neck kinematics and neck dissability index. An approach based on functional regression. Scientific Reports. 14(1). 10.1038/s41598-023-50562-x 10.1038/s41598-023-50562-x PMC10761888 38167615

[17] Ramsay J, Silverman BW (2005) Functional data analysis (Springer Series in Statistics). Springer, p 430

[18] Dannenmaier J, Kaltenbach C, Kölle T, Krischak G (2020) Application of functional data analysis to explore movements: walking, running and jumping - a systematic review. Gait & Posture. 77:182–189. 10.1016/j.gaitpost.2020.02.002 32058281 10.1016/j.gaitpost.2020.02.002

[19] Warmenhoven J, Harrison A, Robinson MA, Vanrenterghem J, Bargary N, Smith R, Cobley S, Draper C, Donnelly C, Pataky T (2018) A force profile analysis comparison between functional data analysis, statistical parametric mapping and statistical non-parametric mapping in on-water single sculling. J Sci Med Sport 21(10):1100–1105. 10.1016/j.jsams.2018.03.009 29650339 10.1016/j.jsams.2018.03.009

[20] Liew BXW, Rugamer D, Stocker A, De Nunzio AM (2020) Classifying neck pain status using scalar and functional biomechanical variables – development of a method using functional data boosting. Gait & Posture. 76:146–150. 10.1016/j.gaitpost.2019.12.008 31855805 10.1016/j.gaitpost.2019.12.008

[21] Dewig DR, Evans-Pickett A, Pietrosimone BG, Blackburn JT (2023) Comparison of discrete and continuous analysis approaches for evaluating gait biomechanics in individuals with anterior cruciate ligament reconstruction. Gait & Posture. 100:261–267. 10.1016/j.gaitpost.2023.01.012 36682319 10.1016/j.gaitpost.2023.01.012

[22] Mazlan MH, Osman NAA, Abas WABW (2011) Hip 3D joint mechanics analysis of normal and obese individuals’ gait. Springer, pp 161–166. 10.1007/978-3-642-21729-6_43

[23] Pataky TC, Vanrenterghem J, Robinson MA (2016) The probability of false positives in zero-dimensional analyses of one-dimensional kinematic, force and emg trajectories. J Biomech 49(9):1468–1476. 10.1016/j.jbiomech.2016.03.032 27067363 10.1016/j.jbiomech.2016.03.032

[24] Olsen NL, Pini A, Vantini S (2021) False discovery rate for functional data. TEST 30(3):784–809. 10.1007/s11749-020-00751-x

[25] Vernon H (2008) The neck disability index: state-of-the-art, 1991–2008. J Manipulative Physiol Ther 31(7):491–502. 10.1016/j.jmpt.2008.08.006 18803999 10.1016/j.jmpt.2008.08.006

[26] Smyth GK (2004) Linear models and empirical Bayes methods for assessing differential expression in microarray experiments. Stat Appl Genet Mol Biol 1:3 10.2202/1544-6115.1027 16646809

[27] Ritchie ME, Phipson B, Wu D, Hu Y, Law CW, Shi W, Smyth GK (2015) limma powers differential expression analyses for RNA-sequencing and microarray studies. Nucleic Acids Res 43(7):47. 10.1093/nar/gkv007 10.1093/nar/gkv007 PMC4402510 25605792

[28] Benjamini Y, Hochberg Y (1995) Controlling the false discovery rate: a practical and powerful approach to multiple testing. Journal of the Royal Statistical Society. Series B (Methodological). 57(1):289–300

[29] Serra-Añó P, Venegas W, Page A, Torre M, Aguilar-Rodríguez M, Espí-López G (2023) Immediate effects of a single session of cervical spine manipulation on cervical movement patterns in people with nonspecific neck pain: a randomized controlled trial. J Manipulative Physiol Ther 46(1):17–26. 10.1016/j.jmpt.2023.05.006 37422751 10.1016/j.jmpt.2023.05.006

[30] Page A, Rosario H, Mata V, Atienza C (2009) Experimental analysis of rigid body motion. A vector method to determine finite and infinitesimal displacements from point coordinates. J Mech Des 131(3). 10.1115/1.3066468

[31] Andrade Ortega JA, Delgado Martínez AD, Ruiz RA (2010) Validation of the Spanish version of the neck disability index. Spine 35(4):114–118. 10.1097/brs.0b013e3181afea5d 10.1097/BRS.0b013e3181afea5d 20110848

[32] Page A, Inglés M, Venegas W, Mollà-Casanova S, Serra-Añó P (2023) Effect of non-specific neck pain on the path of the instantaneous axis of rotation of the neck during its flexion-extension movement. Musculoskelet Sci Pract 64:102737. 10.1016/j.msksp.2023.102737 10.1016/j.msksp.2023.102737 36871441

[33] Benjamini Y, Yekutieli D (2001) The control of the false discovery rate in multiple testing under dependency. Ann Stat 29(4):1165–1188

[34] R Core Team: R (2025) A language and environment for statistical computing. R Foundation for Statistical Computing, Vienna, Austria. R Found Stat Comput. https://www.R-project.org/

[35] Ramsay J (2023) FDA: functional data analysis. R package version 6.1.4. https://CRAN.R-project.org/package=fda

[36] Febrero-Bande M, Oviedo de la Fuente M (2012) Statistical computing in functional data analysis: the R package fda.usc. J Stat Softw 51(4):1–28

[37] Ritchie ME, Phipson B, Wu D, Hu Y, Law CW, Shi W, Smyth GK (2015) limma powers differential expression analyses for RNA-sequencing and microarray studies. Nucleic Acids Res 43(7):47. 10.1093/nar/gkv007 10.1093/nar/gkv007 PMC4402510 25605792

[38] Huber W, Carey VJ, Gentleman R, Anders S, Carlson M, Carvalho BS, Bravo HC, Davis S, Gatto L, Girke T, Gottardo R, Hahne F, Hansen KD, Irizarry RA, Lawrence M, Love MI, MacDonald J, Obenchain V, Ole’s AK, Pag‘es H, Reyes A, Shannon P, Smyth GK, Tenenbaum D, Waldron L, Morgan M (2015) Orchestrating high-throughput genomic analysis with Bioconductor. Nat Methods 12(2):115–121 10.1038/nmeth.3252 PMC4509590 25633503

[39] Huber W, Carey VJ, Gentleman R, Anders S, Carlson M, Carvalho BS, Bravo HC, Davis S, Gatto L, Girke T, Gottardo R, Hahne F, Hansen KD, Irizarry RA, Lawrence M, Love MI, MacDonald J, Obenchain V, Ole’s AK, Pag‘es H, Reyes A, Shannon P, Smyth GK, Tenenbaum D, Waldron L, Morgan M (2015) Orchestrating high-throughput genomic analysis with Bioconductor. Nat Methods 12(2):115–121 10.1038/nmeth.3252 PMC4509590 25633503

[40] Baydal-Bertomeu JM, Page AF, Belda-Lois JM, Garrido-Jaén D, Prat JM (2011) Neck motion patterns in whiplash-associated disorders: quantifying variability and spontaneity of movement. Clin Biomech 26(1):29–34. 10.1016/j.clinbiomech.2010.08.008 10.1016/j.clinbiomech.2010.08.008 20858573

[41] Magaña LC, Murati S, Riffitts M, Harrison C, Harris A, Sowa G, Johnson JT, Bell K, Nilsen M (2021) Subjective and objective measures in assessing neck disability and pain in head and neck cancer. Laryngoscope 131(9):2015–2022. 10.1002/lary.29488 33656195 10.1002/lary.29488

[42] Pinheiro CF, Oliveira AS, Will-Lemos T, Florencio LL, Fernández-de-las-Peñas C, Dach F, Bevilaqua-Grossi D (2021) Neck active movements assessment in women with episodic and chronic migraine. J Clin Med 10(17):3805. 10.3390/jcm10173805 34501252 10.3390/jcm10173805 PMC8432227

[43] Bahat HS, Igbariya M, Quek J, Treleaven J (2016) Cervical kinematics of fast neck motion across age. J Novel Physiotherapies 6(5). 10.4172/2165-7025.1000306

[44] Liengswangwong W, Lertviboonluk N, Yuksen C, Jamkrajang P, Limroongreungrat W, Mongkolpichayaruk A, Jenpanitpong C, Watcharakitpaisan S, Palee C, Reechaipichitkool P, Thaipasong S (2024) Validity of inertial measurement unit (IMU sensor) for measurement of cervical spine motion, compared with eight optoelectronic 3d cameras under spinal immobilization devices. Med Devices: Evid Res 17:261–269. 10.2147/mder.s475166 10.2147/MDER.S475166 PMC11268762 39050910

[45] Trinidad-Fernández M, Bossavit B, Salgado-Fernández J, Abbate-Chica S, Fernández-Leiva AJ, Cuesta-Vargas AI (2023) Head-mounted display for clinical evaluation of neck movement validation with meta quest 2. Sensors 23(6):3077. 10.3390/s23063077 36991788 10.3390/s23063077 PMC10056752

