# Supplementary material for: Non-specific neck pain evaluation using functional linear models with the limma correction
Source: Med Biol Eng Comput. 2025 Jul 8;63(11):3435–46. doi: 10.1007/s11517-025-03400-3 (PMC12634767; doi:10.1007/s11517-025-03400-3)
Supplement: Supplementary file 1 — (pdf 654 KB) [file 11517_2025_3400_MOESM1_ESM.pdf]

Supplementary material: Non-specific neck pain evaluation using functional linear models with the limma correction

Elisa Aragón-Basanta

Table of contents

Basics

Packages and data

First the packages used later are loaded.

```
library(fda.usc)
library(fda)
library(ggplot2)
library(Biobase)
library(limma)
library(patchwork)
library(ggpubr)
library(GSEAlm)
library(latex2exp)
```

The data sets are loaded.

```
rm(list=ls())
load("data_limma.Rda")

dfi=data_limma$dfi   # angular velocity
d2fi=data_limma$d2fi # angular aceleration
ndi=data_limma$ndi    # Neck Dissability Index (NDI)
```

```
gender=data_limma$gender
age=data_limma$age
long_neck=data_limma$long_neck
```

An outlier is removed and no longer considered in the study.

```
dfi=dfi[-40,]
d2fi=d2fi[-40,]
ndi=ndi[-40]
long_neck=long_neck[-40]
gender=gender[-40]
age=age[-40]
```

The number of observations is calculated.

```
N=length(dfi[,1])
```

The times where the functions will be evaluated are defined and the time increment evaluated.

```
t=seq(0,1,length.out=101)
dt=t[2]-t[1]
```

Evaluating at common times

A functional data object is constructed for the **velocity**.

```
dfi_d = fdata(dfi, argvals = t)   # velocity
ejes_df=list("normalized time","Angular velocity",
             "Angular velocity (º/s)")
dfi_basis=create.bspline.basis(nbasis=40)
dfi_d = Data2fd(argvals = t, y=t(dfi), basisobj = dfi_basis,
                fdnames = ejes_df)
```

A functional data object is constructed for the **acceleration**.

```
d2fi_d = fdata(d2fi, argvals = t)   # acceleration
ejes_d2f=list("normalized time","Angular acceleration",
             "Angular acceleration (º/s^2)")
d2fi_basis=create.bspline.basis(nbasis=40)
d2fi_d = Data2fd(argvals = t, y=t(d2fi), basisobj = d2fi_basis,
```

```
                  fdnames = ejes_d2f)
```

The two functional data objects previously constructed are evaluated at a common set of times.

```
ntimes = 100
t1 = seq(0,1,length.out=ntimes)
sense = rep(1:2,each=ntimes/2)
exprs.dfi = eval.fd(t1,dfi_d)
exprs.d2fi = eval.fd(t1,d2fi_d)
```

The functional mean of velocity is calculated and evaluated at the previously chosen times.

```
m.dfi_d=mean.fd(dfi_d)
m.dfi=eval.fd(m.dfi_d,t1)
```

The functional mean of acceleration is calculated and evaluated at the previously chosen times.

```
m.d2fi_d=mean.fd(d2fi_d)
m.d2fi=eval.fd(m.d2fi_d,t1)
```

ExpressionSet

We are going to use packages from Bioconductor and the class `ExpressionSet` is used to manage the functional data.

```
NDI = data.frame(ndi)
colnames(exprs.dfi) = 1:nrow(NDI)
colnames(exprs.d2fi) = 1:nrow(NDI)
metadatos = data.frame(labelDescription= c("ndi","long_neck","gender","age"))
datosfenotipo = new("AnnotatedDataFrame",
                    data = data.frame(ndi,long_neck,gender,age),
                    varMetadata = metadatos)
y.dfi  = new("ExpressionSet",exprs=exprs.dfi,phenoData = datosfenotipo)
y.d2fi  = new("ExpressionSet",exprs=exprs.d2fi,phenoData = datosfenotipo)
```

Velocity

Comparing Benjamini-Hochberg (Benjamini and Hochberg 1995) and Benjamini-Yekutieli (Benjamini and Yekutieli 2001) corrections

The p-values using the **limma** method are calculated for the velocity.

```
## Making the model matrix
design.dfi = model.matrix(~ pData(y.dfi)$ndi + pData(y.dfi)$long_neck +
                            pData(y.dfi)$gender + pData(y.dfi)$age)
colnames(design.dfi) = c("intercept","ndi","long_neck","gender", "age")
## Fitting the linear models
fit.dfi = limma::lmFit(y.dfi,design.dfi)
## Applying the empirical Bayes procedure
fit1.dfi = eBayes(fit.dfi)
```

The adjusted p-values using the Benjamini-Hochberg correction are evaluated.

```
p1.dfi.BH = p.adjust(fit1.dfi$p.value[,2],method="BH")
p2.dfi.BH = p.adjust(fit1.dfi$p.value[,3],method="BH")
p3.dfi.BH = p.adjust(fit1.dfi$p.value[,4],method="BH")
p4.dfi.BH = p.adjust(fit1.dfi$p.value[,5],method="BH")
```

The adjusted p-values using the Benjamini-Yekutieli correction are evaluated.

```
p1.dfi.BY = p.adjust(fit1.dfi$p.value[,2],method="BY")
p2.dfi.BY = p.adjust(fit1.dfi$p.value[,3],method="BY")
p3.dfi.BY = p.adjust(fit1.dfi$p.value[,4],method="BY")
p4.dfi.BY = p.adjust(fit1.dfi$p.value[,5],method="BY")
```

A linear model is fitted for each time using `GSEAlm::lmPerGene`.

```
lm1.dfi = lmPerGene(y.dfi,~ndi+long_neck+gender+age)
```

The raw p-values where any adjustment is performed are evaluated.

```
p.pap.dfi=matrix(nrow = ntimes, ncol = 4)
for (i in 1:4)
  p.pap.dfi[,i]=2*(1-pt(abs(lm1.dfi$tstat[i+1,]),df=50))
```

NDI

The different p-value functions are plotted. The lowest values are the raw p-values, then the adjusted limma p-values with the Benjamini-Hochberg correction and, finally, the adjusted limma p-values with the Benjamini-Yekutieli correction.

```
p.type=rep(c("1_Raw p-value", "2_Adjusted limma BH p-value",
             "3_Adjusted limma BY p-value"),each=ntimes)
dfp1 = rbind(cbind(t1,p.pap.dfi[,1]), cbind(t1,p1.dfi.BH), cbind(t1,p1.dfi.BY))
dfp1 = data.frame(dfp1, as.factor(p.type))
names(dfp1) = c("velocity", "p_value", "Type")
(plot1.dfi = ggplot(dfp1,aes(x=velocity,y=p_value,color=p.type))+geom_line()+
    geom_abline(intercept=.05,slope=0, linetype = 2) +
    geom_abline(intercept=.1,slope=0,linetype = 2, color="blue") + ylim(0,0.25) +
    labs(title="p-value for NDI", x="normalized time", y="p-value") +
    theme(legend.title = element_blank()) +
    scale_color_manual(values = c("#8B8989", "black","red")))
```

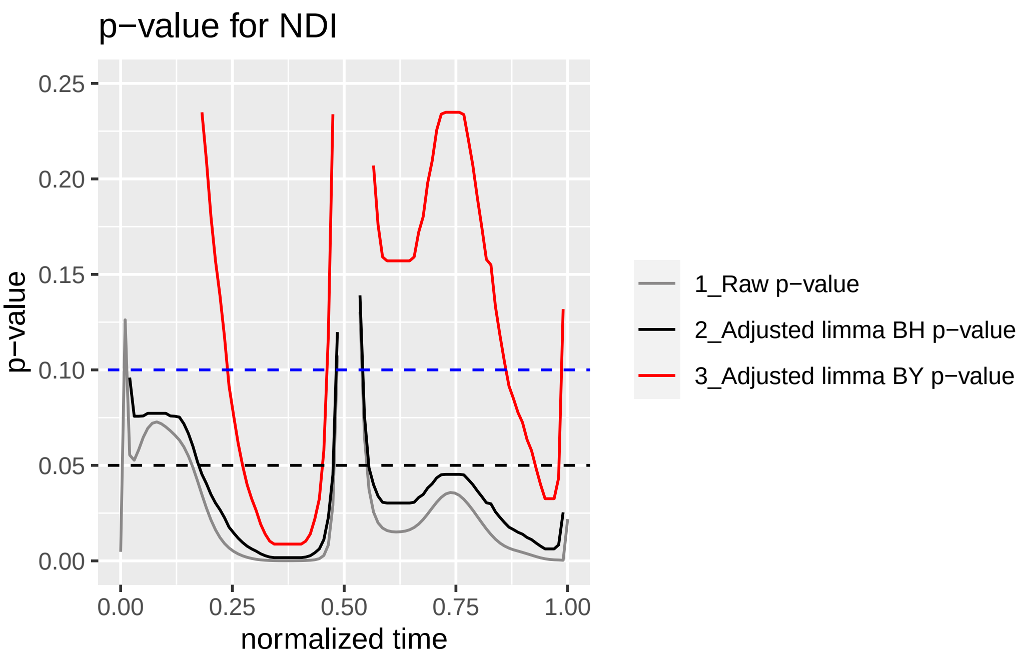

Neck length

```
dfp2 = rbind(cbind(t1,p.pap.dfi[,2]), cbind(t1,p2.dfi.BH), cbind(t1,p2.dfi.BY))
dfp2 = data.frame(dfp2, as.factor(p.type))
names(dfp2) = c("velocity", "p_value", "Type")
(plot2.dfi = ggplot(dfp2,aes(x=velocity,y=p_value,color=p.type))+geom_line()+
    geom_abline(intercept=.05,slope=0, linetype = 2) +
    geom_abline(intercept=.1,slope=0,linetype = 2, color="blue") +
    labs(title="p-value for Neck length", x="normalized time", y="p-value") +
    theme(legend.title = element_blank())+
    scale_color_manual(values = c("#8B8989","black", "red")))
```

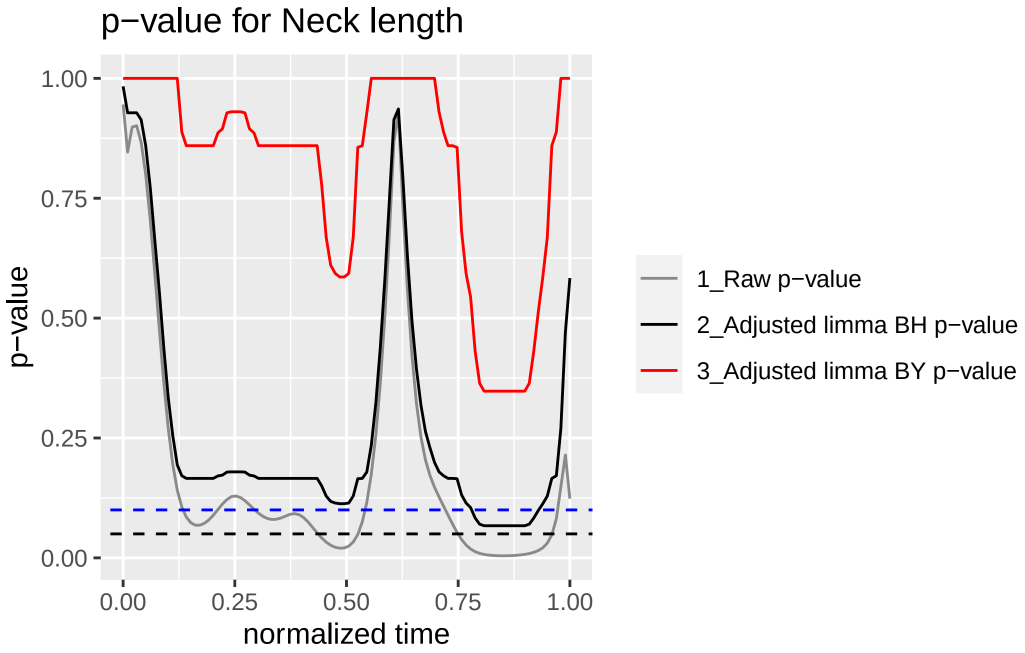

Gender

```
dfp3 = rbind(cbind(t1,p.pap.dfi[,3]), cbind(t1,p3.dfi.BH), cbind(t1,p3.dfi.BY))
dfp3 = data.frame(dfp3, as.factor(p.type))
names(dfp3) = c("velocity", "p_value", "Type")
(plot3.dfi = ggplot(dfp3,aes(x=velocity,y=p_value,color=p.type)) + geom_line() +
     geom_abline(intercept=.05,slope=0,linetype = 2) +
     geom_abline(intercept=.1,slope=0,linetype = 2, color="blue") +
     labs(title="p-value for Gender", x="normalized time", y="p-value") +
```

```
    theme(legend.title = element_blank())+
    scale_color_manual(values = c( "#8B8989", "black","red")))
```

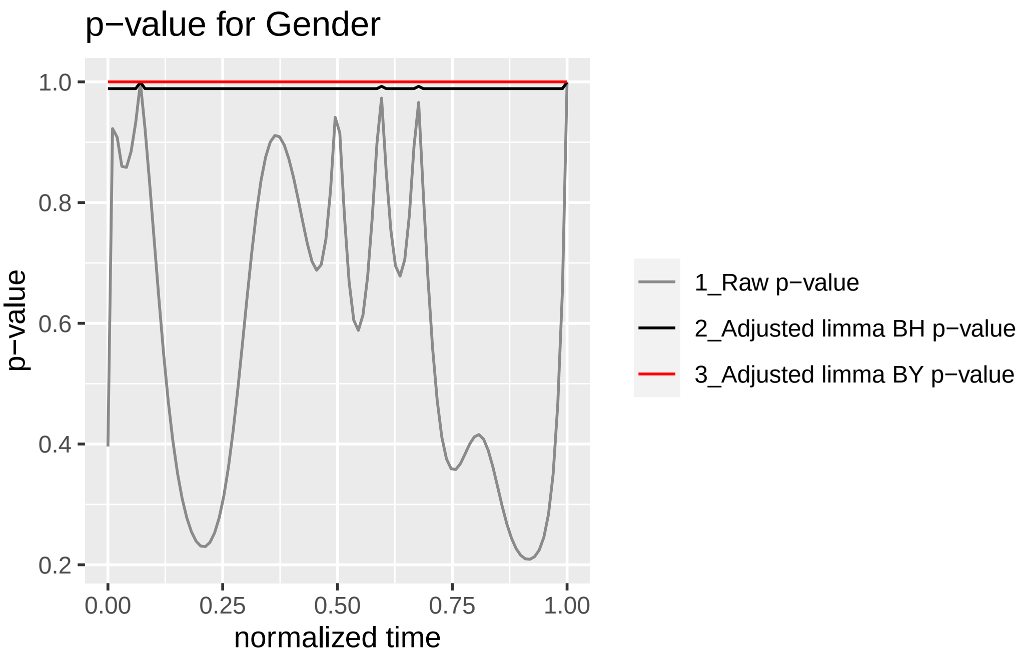

Age

```
dfp4 = rbind(cbind(t1,p.pap.dfi[,4]), cbind(t1,p4.dfi.BH), cbind(t1,p4.dfi.BY))
dfp4 = data.frame(dfp4, as.factor(p.type))
names(dfp4) = c("velocity", "p_value", "Type")
(plot4.dfi = ggplot(dfp4,aes(x=velocity,y=p_value,color=p.type)) +
    geom_line()+ geom_abline(intercept=.05,slope=0, linetype = 2) +
    geom_abline(intercept=.1,slope=0,linetype = 2, color="blue") + ylim(0,0.25)  +
    labs(title="p-value for Age", x="normalized time", y="p-value") +
    theme(legend.title = element_blank())+
    scale_color_manual(values = c("#8B8989","black" ,"red")))
```

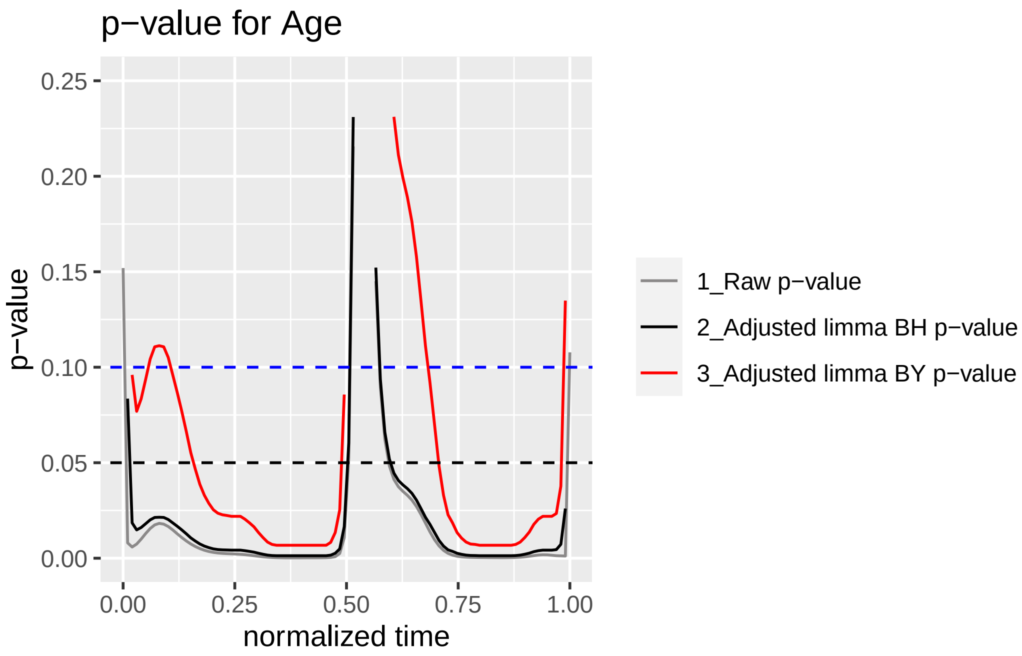

A global plot

The four previous plots are displayed jointly.

```
plot.dfi = ggarrange(plot1.dfi, plot2.dfi, plot3.dfi, plot4.dfi,
                     common.legend = TRUE, legend = "bottom")

annotate_figure(plot.dfi, top = text_grob("Angular velocity",
                                          face = "bold", size = 14))
```

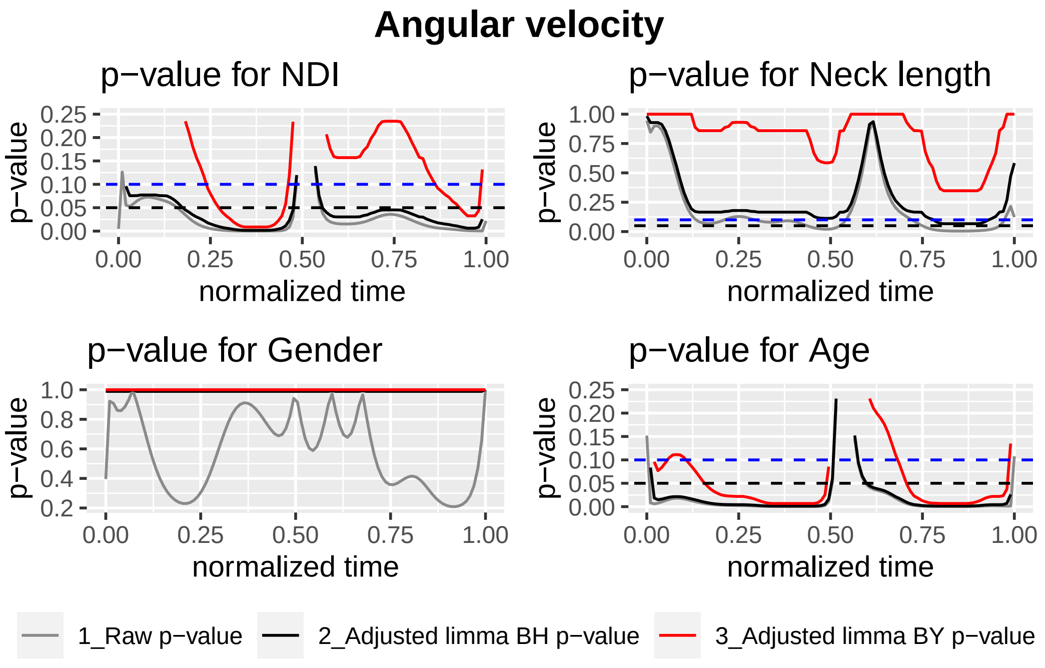

Acceleration

Calculating the p-values

```
## Fitting the models
design.d2fi = model.matrix(~ pData(y.d2fi)$ndi + pData(y.d2fi)$long_neck +
                             pData(y.d2fi)$gender + pData(y.d2fi)$age)
colnames(design.d2fi) = c("intercept","ndi","long_neck","gender", "age")
fit.d2fi = limma::lmFit(y.d2fi,design.d2fi)
fit1.d2fi = eBayes(fit.d2fi)

## Calculating the adjusted p-values with Benjamini-Hochberg correction
p1.d2fi.BH = p.adjust(fit1.d2fi$p.value[,2],method="BH")
p2.d2fi.BH = p.adjust(fit1.d2fi$p.value[,3],method="BH")
p3.d2fi.BH = p.adjust(fit1.d2fi$p.value[,4],method="BH")
p4.d2fi.BH = p.adjust(fit1.d2fi$p.value[,5],method="BH")

## Calculating the adjusted p-values with Benjamini-Yekutieli correction
p1.d2fi.BY = p.adjust(fit1.d2fi$p.value[,2],method="BY")
p2.d2fi.BY = p.adjust(fit1.d2fi$p.value[,3],method="BY")
p3.d2fi.BY = p.adjust(fit1.d2fi$p.value[,4],method="BY")
```

```r
p4.d2fi.BY = p.adjust(fit1.d2fi$p.value[,5],method="BY")

## Linear models at each time using lmPerGene
lm1.d2fi = lmPerGene(y.d2fi,~ndi+long_neck+gender+age)

## Calculating the raw p-values
p.pap.d2fi=matrix(nrow = ntimes, ncol = 4)
for (i in 1:4) p.pap.d2fi[,i]=2*(1-pt(abs(lm1.d2fi$tstat[i+1,]),df=50))

## PLOTS
## NDI
p.type=rep(c("1_Raw p-value", "2_Adjusted limma BH p-value",
             "3_Adjusted limma BY p-value"), each=ntimes)
dfp1 = rbind(cbind(t1,p.pap.d2fi[,1]), cbind(t1,p1.d2fi.BH), cbind(t1,p1.d2fi.BY))
dfp1 = data.frame(dfp1, as.factor(p.type))
names(dfp1) = c("angle", "p_value", "Type")
(plot1.d2fi = ggplot(dfp1,aes(x=angle,y=p_value,color=p.type))+geom_line()+
    geom_abline(intercept=.05,slope=0, linetype = 2) +
    geom_abline(intercept=.1,slope=0,linetype = 2, color="blue") + ylim(0,0.25) +
    labs(title="p-value for NDI", x="normalized time", y="p-value") +
    theme(legend.title = element_blank()) +
    scale_color_manual(values = c("#8B8989", "black","red")))
```

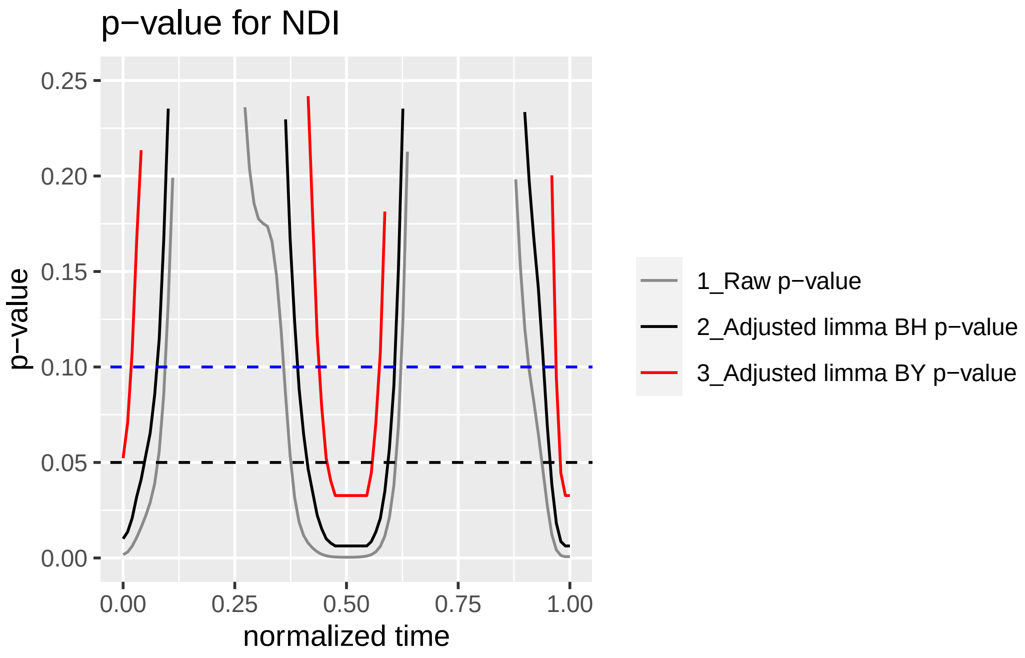

```r
# Neck length
dfp2 = rbind(cbind(t1,p.pap.d2fi[,2]), cbind(t1,p2.d2fi.BH), cbind(t1,p2.d2fi.BY))
dfp2 = data.frame(dfp2, as.factor(p.type))
names(dfp2) = c("acceleration", "p_value", "Type")

(plot2.d2fi = ggplot(dfp2,aes(x=acceleration,y=p_value,color=p.type))+geom_line()+
    geom_abline(intercept=.05,slope=0, linetype = 2) +
    geom_abline(intercept=.1,slope=0,linetype = 2, color="blue") +
    labs(title="p-value for Neck length", x="normalized time", y="p-value") +
    theme(legend.title = element_blank())+
    scale_color_manual(values = c("#8B8989","black", "red")))
```

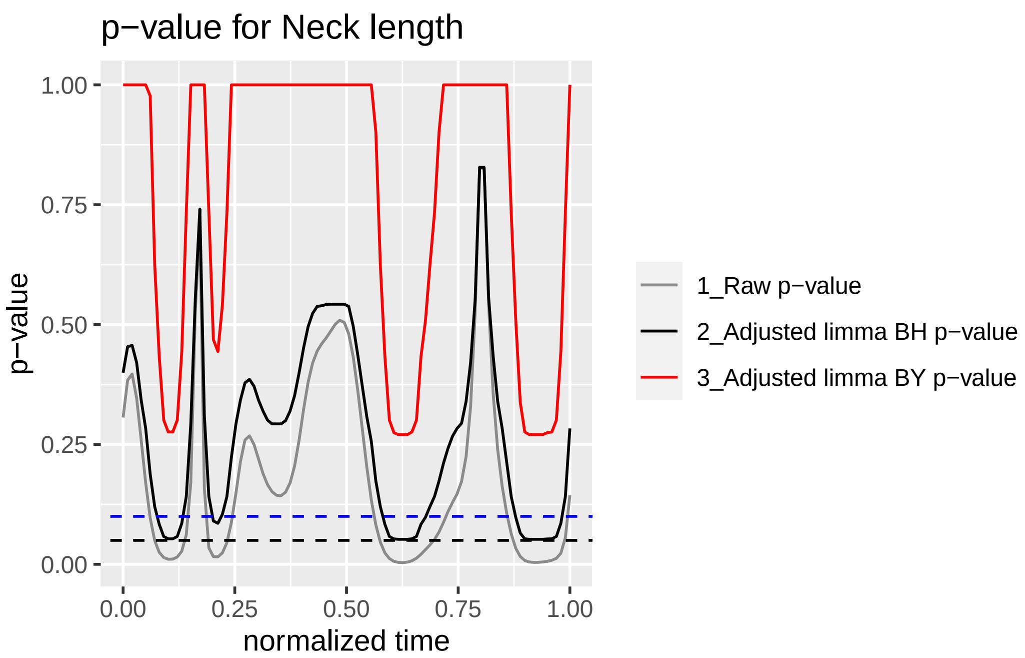


```r
# Gender
dfp3 = rbind(cbind(t1,p.pap.d2fi[,3]), cbind(t1,p3.d2fi.BH), cbind(t1,p3.d2fi.BY))
dfp3 = data.frame(dfp3, as.factor(p.type))
names(dfp3) = c("acceleration", "p_value", "Type")
(plot3.d2fi = ggplot(dfp3,aes(x=acceleration,y=p_value,color=p.type))+geom_line()+
    geom_abline(intercept=.05,slope=0, linetype = 2) +
    geom_abline(intercept=.1,slope=0,linetype = 2, color="blue") +
    labs(title="p-value for Gender", x="normalized time", y="p-value") +
    theme(legend.title = element_blank())+
    scale_color_manual(values = c( "#8B8989", "black","red")))
```

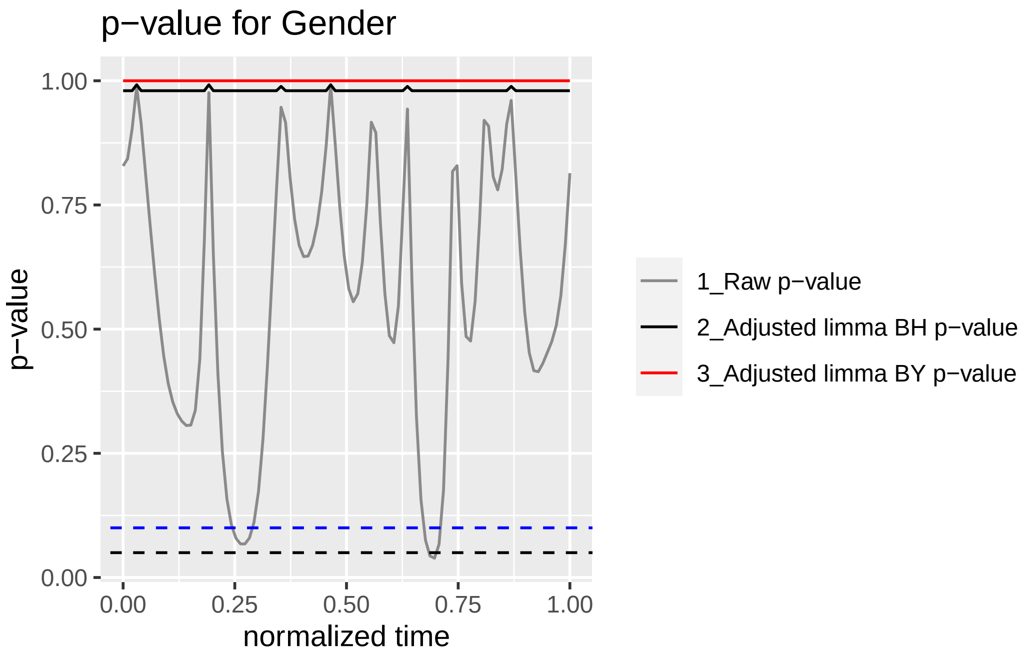


```
# Age
dfp4 = rbind(cbind(t1,p.pap.d2fi[,4]), cbind(t1,p4.d2fi.BH), cbind(t1,p4.d2fi.BY))
dfp4 = data.frame(dfp4, as.factor(p.type))
names(dfp4) = c("acceleration", "p_value", "Type")
(plot4.d2fi = ggplot(dfp4,aes(x=acceleration,y=p_value,color=p.type))+
    geom_line()+ geom_abline(intercept=.05,slope=0, linetype = 2)  +
    geom_abline(intercept=.1,slope=0,linetype = 2, color="blue") +
    labs(title="p-value for Age", x="normalized time", y="p-value") +
    theme(legend.title = element_blank())+
    scale_color_manual(values = c("#8B8989","black" ,"red")))
```

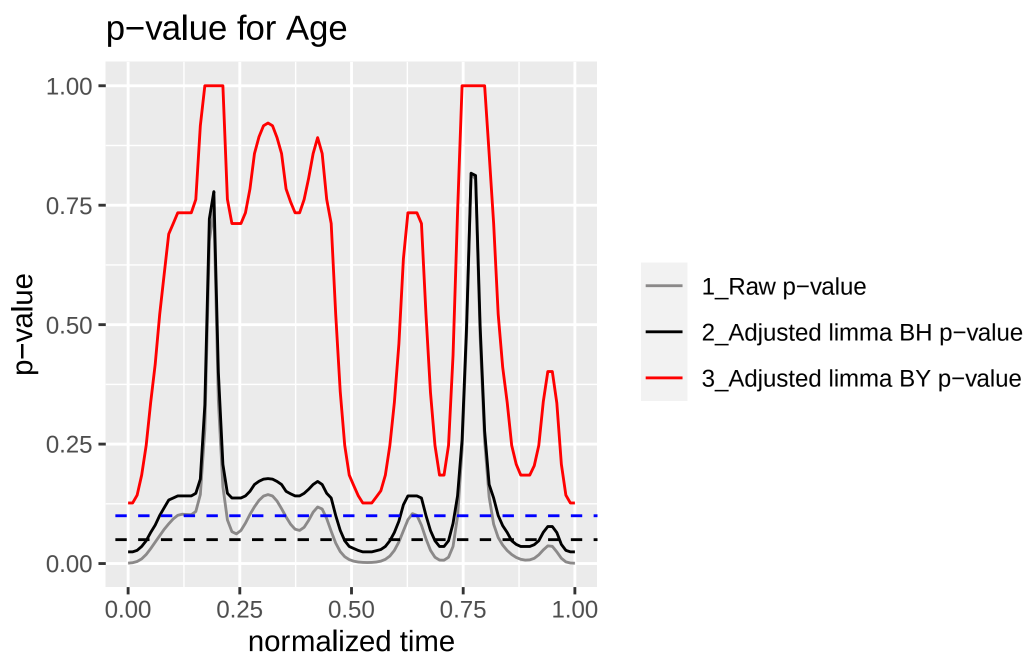

Finally, a global plot as before.

```
plot.d2fi = ggarrange(plot1.d2fi, plot2.d2fi, plot3.d2fi, plot4.d2fi,
                      common.legend = TRUE, legend = "bottom")
annotate_figure(plot.d2fi, top = text_grob("Angular acceleration",
                                           face = "bold", size = 14))
```

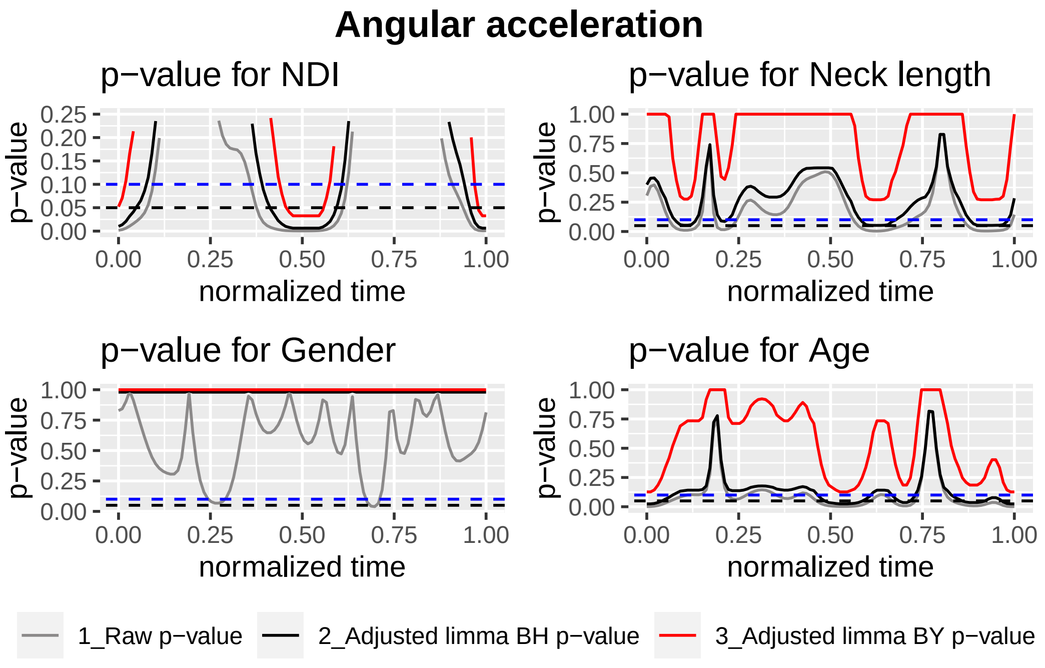

From both the velocity and angular acceleration plots, it can be seen that a correction of the raw p-values is necessary. The Benjamini-Yekutieli correction is more conservative than the Benjamini-Hochberg correction, as seen from the velocity and acceleration p-value plots. In angular velocity, neck length, and gender are not significant predictors, while NDI and age are significant using both corrections. Although the BY correction is more conservative and somewhat reduces the areas where the p-value is significant, these areas are similar using both corrections. A similar situation occurs with angular acceleration, although in this case, only NDI would be significant with the BY correction. At the same time, neck length and age would also be significant with the BH correction in certain areas.

Evaluation of the number of times

Velocity

First, we choose 1000 points.

```
# 1000 points
ntimes = 1000
t1.1000 = seq(0,1,length.out=ntimes)
sense = rep(1:2,each=ntimes/2)
exprs.dfi.1000 = eval.fd(t1.1000,dfi_d)
```

```r
# mean of velocity
m.dfi.1000=eval.fd(m.dfi_d,t1.1000)

y.dfi.1000  = new("ExpressionSet",exprs=exprs.dfi.1000,phenoData = datosfenotipo)

## Limma model
design.dfi.1000 = model.matrix(~ pData(y.dfi.1000)$ndi + pData(y.dfi.1000)$long_neck
                               + pData(y.dfi.1000)$gender + pData(y.dfi.1000)$age)
colnames(design.dfi.1000) = c("intercept","ndi","long_neck","gender", "age")
fit.dfi.1000 = limma::lmFit(y.dfi.1000,design.dfi.1000)
fit1.dfi.1000 = eBayes(fit.dfi.1000)

# Adjusted p-value of limma model with Benjamini-Hochberg correction
p1.dfi.1000 = p.adjust(fit1.dfi.1000$p.value[,2],method="BH")
p2.dfi.1000 = p.adjust(fit1.dfi.1000$p.value[,3],method="BH")
p3.dfi.1000 = p.adjust(fit1.dfi.1000$p.value[,4],method="BH")
p4.dfi.1000 = p.adjust(fit1.dfi.1000$p.value[,5],method="BH")

## 10.000 points
ntimes = 10000
t1.10000 = seq(0,1,length.out=ntimes)
sense = rep(1:2,each=ntimes/2)
exprs.dfi.10000 = eval.fd(t1.10000,dfi_d)

# Mean of velocity
m.dfi=eval.fd(m.dfi_d,t1.10000)

y.dfi.10000  = new("ExpressionSet",exprs=exprs.dfi.10000,phenoData = datosfenotipo)

## Limma model
design.dfi.10000 = model.matrix(~ pData(y.dfi.10000)$ndi +
                  pData(y.dfi.10000)$long_neck + pData(y.dfi.10000)$gender +
                  pData(y.dfi.10000)$age)
colnames(design.dfi.10000) = c("intercept","ndi","long_neck","gender", "age")
fit.dfi.10000 = limma::lmFit(y.dfi.10000,design.dfi.10000)
fit1.dfi.10000 = eBayes(fit.dfi.10000)

# Adjusted p-value of limma model with Benjamini-Hochberg correction
p1.dfi.10000 = p.adjust(fit1.dfi.10000$p.value[,2],method="BH")
p2.dfi.10000 = p.adjust(fit1.dfi.10000$p.value[,3],method="BH")
p3.dfi.10000 = p.adjust(fit1.dfi.10000$p.value[,4],method="BH")
```

```
p4.dfi.10000 = p.adjust(fit1.dfi.10000$p.value[,5],method="BH")

# Plot
par(mar = c(2, 2, 2, 0.5), mfrow=c(2,2))
# NDI
plot(t1, p1.dfi.BH, type="l", ylim = c(0, 0.1), main = "NDI")
lines(t1.1000, p1.dfi.1000, col="red")
lines(t1.10000, p1.dfi.10000, col="blue")
legend("bottomleft",legend=c("100 points","1.000 points", "10.000 points"),
       col=c("black","red", "blue"),cex = 0.5,lty =  c("solid","solid","solid"))

# Neck lenght
plot(t1, p2.dfi.BH, type="l", ylim = c(0, 0.3), main = "Neck length")
lines(t1.1000, p2.dfi.1000, col="red")
lines(t1.10000, p2.dfi.10000, col="blue")
legend("bottomleft",legend=c("100 points","1.000 points", "10.000 points"),
       col=c("black","red", "blue"),cex = 0.5,lty =  c("solid","solid","solid"))

# Gender
plot(t1, p3.dfi.BH, type="l", ylim = c(0.8,1),main = "Gender")
lines(t1.1000, p3.dfi.1000, col="red")
lines(t1.10000, p3.dfi.10000, col="blue")
legend("bottomright",legend=c("100 points","1.000 points", "10.000 points"),
       col=c("black","red", "blue"),cex = 0.5,lty =  c("solid","solid","solid"))

# Age
plot(t1, p4.dfi.BH, type="l", ylim = c(0, 0.1), main = "Age")
lines(t1.1000, p4.dfi.1000, col="red")
lines(t1.10000, p4.dfi.10000, col="blue")
legend("topright",legend=c("100 points","1.000 points", "10.000 points"),
       col=c("black","red", "blue"),cex = 0.5,lty =  c("solid","solid","solid"))
```

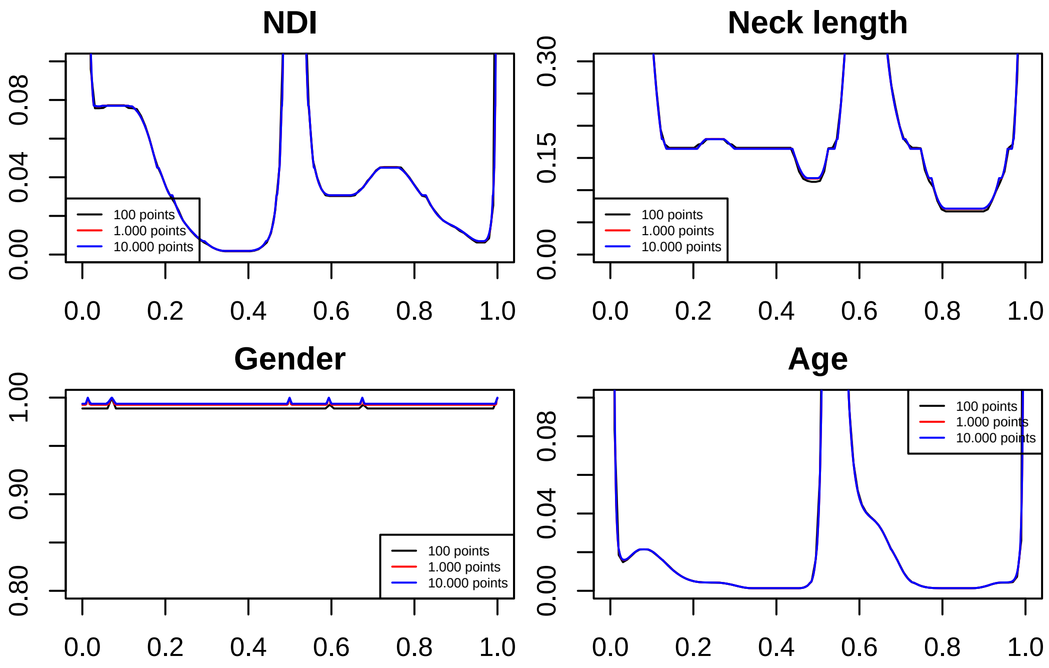

Acceleration

```
# 1000 points
exprs.d2fi.1000 = eval.fd(t1.1000,d2fi_d)

# mean of acceleration
m.d2fi.1000=eval.fd(m.d2fi_d,t1.1000)

y.d2fi.1000  = new("ExpressionSet",exprs=exprs.d2fi.1000,phenoData = datosfenotipo)

## Limma model
design.d2fi.1000 =
    model.matrix(~ pDaTa(y.d2fi.1000)$ndi + pData(y.d2fi.1000)$long_neck +
                   pData(y.d2fi.1000)$gender + pData(y.d2fi.1000)$age)
colnames(design.d2fi.1000) = c("intercept","ndi","long_neck","gender", "age")
fit.d2fi.1000 = limma::lmFit(y.d2fi.1000,design.d2fi.1000)
fit1.d2fi.1000 = eBayes(fit.d2fi.1000)

# Adjusted p-value of limma model with Benjamini-Hochberg correction
p1.d2fi.1000 = p.adjust(fit1.d2fi.1000$p.value[,2],method="BH")
p2.d2fi.1000 = p.adjust(fit1.d2fi.1000$p.value[,3],method="BH")
p3.d2fi.1000 = p.adjust(fit1.d2fi.1000$p.value[,4],method="BH")
```

```r
p4.d2fi.1000 = p.adjust(fit1.d2fi.1000$p.value[,5],method="BH")

## 10.000 points
exprs.d2fi.10000 = eval.fd(t1.10000,d2fi_d)

# Mean of acceleration
m.d2fi=eval.fd(m.d2fi_d,t1.10000)

y.d2fi.10000  = new("ExpressionSet",exprs=exprs.d2fi.10000,phenoData = datosfenotipo)

## Limma model
design.d2fi.10000 = model.matrix(~ pData(y.d2fi.10000)$ndi +
                  pData(y.d2fi.10000)$long_neck + pData(y.d2fi.10000)$gender +
                  pData(y.d2fi.10000)$age)
colnames(design.d2fi.10000) = c("intercept","ndi","long_neck","gender", "age")
fit.d2fi.10000 = limma::lmFit(y.d2fi.10000,design.d2fi.10000)
fit1.d2fi.10000 = eBayes(fit.d2fi.10000)

# Adjusted p-value of limma model with Benjamini-Hochberg correction
p1.d2fi.10000 = p.adjust(fit1.d2fi.10000$p.value[,2],method="BH")
p2.d2fi.10000 = p.adjust(fit1.d2fi.10000$p.value[,3],method="BH")
p3.d2fi.10000 = p.adjust(fit1.d2fi.10000$p.value[,4],method="BH")
p4.d2fi.10000 = p.adjust(fit1.d2fi.10000$p.value[,5],method="BH")

# Plot
par(mar = c(2, 2, 2, 0.5), mfrow=c(2,2))
# NDI
plot(t1, p1.d2fi.BH, type="l", ylim = c(0, 0.1), main = "NDI")
lines(t1.1000, p1.d2fi.1000, col="red")
lines(t1.10000, p1.d2fi.10000, col="blue")
legend("topright",legend=c("100 points","1.000 points", "10.000 points"),
       col=c("black","red", "blue"),cex = 0.5,lty =  c("solid","solid","solid"))

# Neck lenght
plot(t1, p2.d2fi.BH, type="l", ylim = c(0, 0.3), main = "Neck length")
lines(t1.1000, p2.d2fi.1000, col="red")
lines(t1.10000, p2.d2fi.10000, col="blue")
legend("topright",legend=c("100 points","1.000 points", "10.000 points"),
       col=c("black","red", "blue"),cex = 0.5,lty =  c("solid","solid","solid"))

# Gender
```

```r
plot(t1, p3.d2fi.BH, type="l", ylim = c(0.8,1),main = "Gender")
lines(t1.1000, p3.d2fi.1000, col="red")
lines(t1.10000, p3.d2fi.10000, col="blue")
legend("bottomright",legend=c("100 points","1.000 points", "10.000 points"),
       col=c("black","red", "blue"),cex = 0.5,lty =  c("solid","solid","solid"))

# Age
plot(t1, p4.d2fi.BH, type="l", ylim = c(0, 0.1), main = "Age")
lines(t1.1000, p4.d2fi.1000, col="red")
lines(t1.10000, p4.d2fi.10000, col="blue")
legend("bottomleft",legend=c("100 points","1.000 points", "10.000 points"),
       col=c("black","red", "blue"),cex = 0.5,lty =  c("solid","solid","solid"))
```

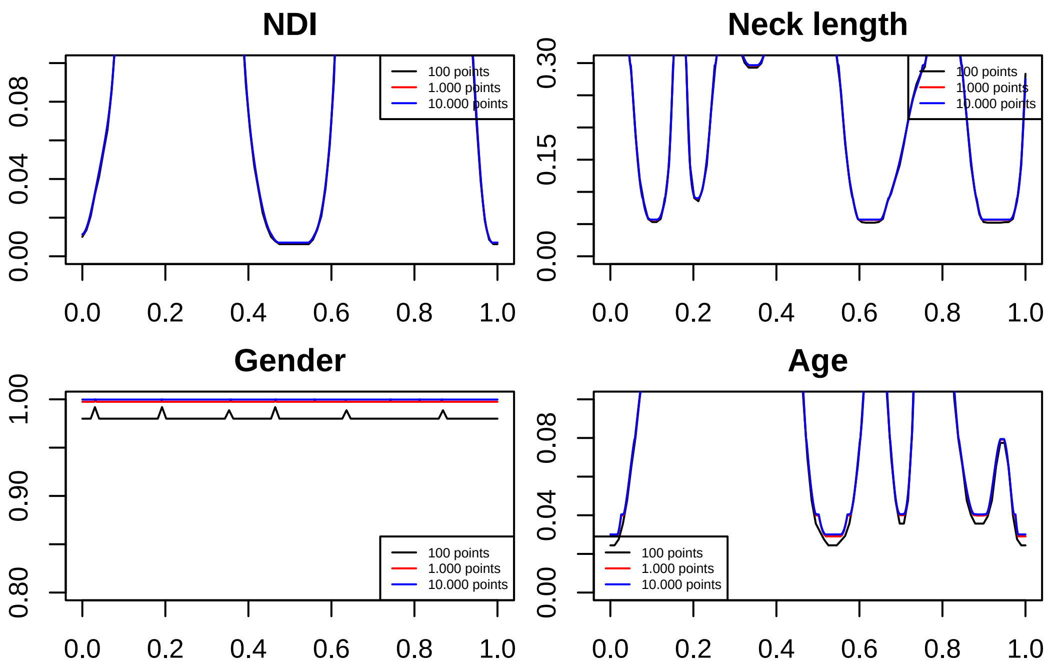

The plots above show the p-values of the limma model with the Benjamini-Hochberg correction, taking 100 (black), 1000 (red), and 10,000 (blue) points. It is demonstrated that the number of points is irrelevant and that the differences obtained in the p-values are negligible for the four predictors considered, both in velocity and acceleration.

Bibliography

Benjamini, Yoav, and Yosef Hochberg. 1995. "Controlling the False Discovery Rate: A Practical and Powerful Approach to Multiple Testing." *Journal of the Royal Statistical Society. Series*

B (Methodological) 57 (1): 289–300. http://www.jstor.org/stable/2346101.

Benjamini, Yoav, and Daniel Yekutieli. 2001. "The Control of the False Discovery Rate in Multiple Testing Under Dependency." *The Annals of Statistics* 29 (4): 1165–88.
